# Alpha-synuclein seeding shows a wide heterogeneity in multiple system atrophy

**DOI:** 10.1186/s40035-022-00283-4

**Published:** 2022-02-07

**Authors:** Ivan Martinez-Valbuena, Naomi P. Visanji, Ain Kim, Heather H. C. Lau, Raphaella W. L. So, Sohaila Alshimemeri, Andrew Gao, Michael A. Seidman, Maria R. Luquin, Joel C. Watts, Anthony E. Lang, Gabor G. Kovacs

**Affiliations:** 1 Tanz Centre for Research in Neurodegenerative Diseases, University of Toronto, Toronto, ON Canada; 2 Edmond J. Safra Program in PD and the Morton and Gloria Shulman Movement Disorders Clinic, Toronto Western Hospital, Toronto, ON Canada; 3 Department of Laboratory Medicine and Pathobiology, University of Toronto, Toronto, ON Canada; 4 Krembil Brain Institute, University Health Network, Toronto, ON Canada; 5 Department of Biochemistry, University of Toronto, Toronto, ON Canada; 6 Laboratory Medicine Program, University Health Network, Toronto, ON Canada; 7 Department of Neurology, Clinica Universidad de Navarra, Pamplona, Navarra Spain; 8 Present Address: Division of Neurology, Department of Medicine, King Saud University, Riyadh, Saudi Arabia

**Keywords:** Alpha-synuclein, Multiple system atrophy, RT-QuIC, Seeding behavior

## Abstract

**Background:**

Multiple system atrophy (MSA) is a neurodegenerative condition characterized by variable combinations of parkinsonism, autonomic failure, cerebellar ataxia and pyramidal features. Although the distribution of synucleinopathy correlates with the predominant clinical features, the burden of pathology does not fully explain observed differences in clinical presentation and rate of disease progression. We hypothesized that the clinical heterogeneity in MSA is a consequence of variability in the seeding activity of α-synuclein both between different patients and between different brain regions.

**Methods:**

The reliable detection of α-synuclein seeding activity derived from MSA using cell-free amplification assays remains challenging. Therefore, we conducted a systematic evaluation of 168 different reaction buffers, using an array of pH and salts, seeded with fully characterized brain homogenates from one MSA and one PD patient. We then validated the two conditions that conferred the optimal ability to discriminate between PD- and MSA-derived samples in a larger cohort of 40 neuropathologically confirmed cases, including 15 MSA. Finally, in a subset of brains, we conducted the first multi-region analysis of seeding behaviour in MSA.

**Results:**

Using our novel buffer conditions, we show that the physicochemical factors that govern the in vitro amplification of α-synuclein can be tailored to generate strain-specific reaction buffers that can be used to reliably study the seeding capacity from MSA-derived α-synuclein. Using this novel approach, we were able to sub-categorize the 15 MSA brains into 3 groups: high, intermediate and low seeders. To further demonstrate heterogeneity in α-synuclein seeding in MSA, we conducted a comprehensive multi-regional evaluation of α-synuclein seeding in 13 different regions from 2 high seeders, 2 intermediate seeders and 2 low seeders.

**Conclusions:**

We have identified unexpected differences in seed-competent α-synuclein across a cohort of neuropathologically comparable MSA brains. Furthermore, our work has revealed a substantial heterogeneity in seeding activity, driven by the PBS-soluble α-synuclein, between different brain regions of a given individual that goes beyond immunohistochemical observations. Our observations pave the way for future subclassification of MSA, which exceeds conventional clinical and neuropathological phenotyping and considers the structural and biochemical heterogeneity of α-synuclein present. Finally, our methods provide an experimental framework for the development of vitally needed, rapid and sensitive diagnostic assays for MSA.

**Supplementary Information:**

The online version contains supplementary material available at 10.1186/s40035-022-00283-4.

## Introduction

Multiple system atrophy (MSA) is a progressive neurodegenerative disorder with clinical presentation of various combinations of parkinsonism, cerebellar and autonomic dysfunction [1]. The term MSA was coined in 1969 to pool previously described neurological entities [2]; however, the major common finding of argyrophilic oligodendrocytic glial cytoplasmic inclusions (GCIs), called Papp-Lantos bodies, contributed significantly to the nosological definition of the disease [3]. The presence of the 140-amino-acid protein, α-synuclein, as a major component of these inclusions linked MSA with Lewy body disorders such as Parkinson’s disease (PD) and dementia with Lewy bodies (DLB) [4]. Collectively, these disorders are now termed synucleinopathies. Lewy body disorders are classically distinguished from MSA by distinct cellular pathology. Although both conditions accumulate α-synuclein in a variety of cell types, MSA is characterized by oligodendrocytic inclusions, while neuronal α-synuclein pathology predominates in Lewy body disorders. In addition, recent studies have uncovered the presence of α-synuclein “polymorphs”, suggestive of different strains as occurs in prion diseases, which might be the basis for the phenotypic diversity found in these conditions. The presence of different strains is hypothesized to dictate the cell-to-cell spreading of pathology and the cellular impact of the pathological α-synuclein in every individual [5, 6]. Consistent with this notion, many experimental findings have indicated that α-synuclein forming GCIs has greater seeding activity compared to Lewy body-associated α-synuclein [7, 8]. Furthermore, recent discoveries using cryo-electron microscopy have shown structural differences between aggregates found in MSA and those in DLB brains [9, 10].

In recent years, the seeded amplification assays have emerged as a reliable method of detecting minute amounts of misfolded disease-associated proteins or seeds such as prion protein, tau and α-synuclein [11]. These assays, also known as Real-Time Quaking-induced Conversion (RT-QuIC) and protein misfolding cyclic amplification (PMCA), exploit the property of self-propagation to amplify and then sensitively detect minute amounts of these protein seeds [12, 13]. Real-time detection of thioflavin T (ThT) fluorescence at multiple time points during the assay permits the measurement of kinetic differences between seeding properties of different samples, providing a highly specific characterization of whether a disease-associated protein is present or not. However, despite the reliability of these assays to consistently detect α-synuclein seeds in Lewy body disorders, using a variety of biological samples [14–17], attempts to detect α-synuclein in MSA have proven challenging. Van Rumund et al*.* found that α-synuclein RT-QuIC was positive in only 6/17 cerebrospinal fluid (CSF) samples in MSA [16], while Rossi et al*.* detected an even lower number of RT-QuIC positive CSF samples in MSA (2/29) [17]. In contrast, using PMCA over a period of 350 h, Shahawanaz et al*.* were able to detect α-synuclein seeding activity in 65/75 MSA CSF samples, but noted that in spite of aggregating faster, MSA CSF and brain samples reached a lower fluorescence plateau than PD CSF and brain samples [18]. These divergent findings are likely the result of different conditions in the reaction buffers used in these studies. Thus, in the present study, we sought to identify the optimal assay conditions that favor MSA seeding activity, by systematically modulating the ionic and cationic compositions and the pH of the α-synuclein RT-QuIC reaction buffer. The effect of ionic composition and pH on α-synuclein aggregation has been widely studied [19], but not in the context of systematic comparison of a spectrum of reaction buffers seeded with brain homogenates from MSA and PD.

## Material and methods

### Human tissue samples

Fifteen subjects with MSA, 15 subjects with PD, 5 controls and 5 subjects with supranuclear progressive palsy were selected from the University Health Network-Neurodegenerative Brain Collection (UHN-NBC, Toronto, Canada) and the Navarrabiomed Brain Bank (Pamplona, Spain) based on a definite neuropathological diagnosis. Age at death, sex and complete neuropathological examination are listed in the Additional file 1: Table S1. Autopsy tissues from human brains were collected with informed consent from patients or their relatives and approval by local institutional review boards. This study was approved by the University Health Network Research Ethics Board (Nr. 20-5258). Prior to inclusion in the study, a systematic neuropathological examination was performed following diagnostic criteria of neurodegenerative conditions and co-pathologies [20]. The contralateral hemisphere was sliced coronally at the time of autopsy and immediately flash frozen and stored at − 80 °C. Using a 4-mm brain tissue punch, a microdissection of the following regions was performed: anterior cingulate cortex, anterior cingulate white matter, frontal cortex, frontal white matter, putamen, globus pallidus, amygdala, hippocampus, temporal cortex, temporal white matter, substantia nigra (SN), pons base, and cerebellar white matter. All the punches were stored in low protein-binding tubes (Eppendorf, Hamburg, Germany), immediately flash frozen and stored at − 80 °C.

### Protein extraction

For the PBS-soluble fraction, 40–50 mg of frozen microdissected tissue was thawed on wet ice and then immediately homogenized in 500 μl of PBS spiked with protease (Roche, Basel, Switzerland) and phosphatase inhibitors (Thermo Scientific, Waltham, MA) in a gentle-MACS Octo Dissociator (Miltenyi BioTec, Auburn, CA). The homogenate was transferred to a 1.5-ml low protein-binding tube (Eppendorf) and centrifuged at 10,000 *g* for 10 min at 4 °C, as previously described [21]. Then, the supernatant was collected and aliquoted in 0.5-ml low protein-binding tubes (Eppendorf) to avoid excessive freeze–thaw cycles. Sarkosyl-insoluble material was extracted using 1 g of frozen brain tissue from three brain regions (cerebellum, putamen and frontal cortex) of individuals with MSA, as previously described [9]. Bicinchoninic acid protein (BCA) assay (Thermo Scientific) was performed to determine the total protein concentrations of all aliquots.

### Histological analysis

Formalin-fixed paraffin-embedded tissue sections of 4 µm thick containing the 13 anatomical regions selected for microdissection (see above) were examined. In addition to Hematoxylin and Eosin-Luxol Fast Blue, mouse monoclonal antibodies for aggregated α-synuclein (5G4; 1:4000; Roboscreen, Leipzig, Germany), nitrated synuclein (Syn514; 1:2000; Biolegend, San Diego, CA), C-terminal truncated x-122 α-synuclein (A15127A; 1:2000; Biolegend) and phospho-α-synuclein (pSyn#64; 1:10,000; FUJIFILM Wako Pure Chemical Corporation, Osaka, Japan) were used for immunohistochemistry. To map the co-pathology, the following mouse monoclonal antibodies were used: anti-tau AT8 (pS202/pT205; 1:1000; Thermo Scientific), anti-phospho-TDP-43 (pS409/410; 1:2000; Cosmo Bio, Tokyo, Japan) and anti-Aβ (6F/3D; 1:50; Dako, Santa Clara, CA). The DAKO EnVision detection kit, Peroxidase/DAB, Rabbit/Mouse (Dako) was used to visualize the antibody staining. For comparison of different α-synuclein antibodies, immunostained sections against nitrated, truncated, phosphorylated, and aggregated (5G4) α-synuclein were scanned using Tissuescope™ and were cropped with the HuronViewer™ (Huron, Saint Jacobs, Canada). Images were taken from the exact same location of the putamen and cerebellum across the different antibodies. Initially, 100 immunoreactive oligodendrocytes from each antibody, region and case with visible nucleus were optically dissected using Photoshop (2021, Adobe, San Jose, CA). Using Image J (NIH, Bethesda, MD), the minimum and maximum areas (px^2^) of the 100 inclusions were recorded. The density of black dots per unit of inclusions was measured. The amounts of aggregated α-synuclein as well as the GCI and neuronal cytoplasmic inclusion (NCI) burden were assessed by α-synuclein immunohistochemistry in the 13 brain regions mentioned above using the 5G4 staining. For semi-quantitative analyses, we used a 4-point scale: 0, absent; 1, mild; 2, moderate; and 3, severe, as previously described [22].

### ELISA

Human α-Synuclein Patho and Total ELISAs kits (Roboscreen, Leipzig, Germany) were used according to the manufacturer’s protocol and as previously described [23].

### SDS-PAGE and immunoblotting

Gel electrophoresis was performed using 4%–12% or 12% Bolt Bis–Tris Plus gels (Thermo Scientific). Proteins were transferred to 0.45-μm polyvinylidene fluoride membranes for 60 min at 35 V. Proteins were crosslinked to the membrane via 0.4% (*v*/*v*) paraformaldehyde incubation in PBS for 30 min at room temperature, with rocking. The membranes were blocked for 60 min at room temperature in blocking buffer (5% [*w*/*v*] skim milk in 1× TBST (TBS and 0.05% [*v*/*v*] Tween-20)) and then incubated overnight at 4 °C with primary antibody directed against amino acids 15–123 of the α-synuclein protein (1:10,000 dilution, ref: 610786, BD Biosciences, Franklin Lakes, NJ) [8, 24], diluted in the blocking buffer. The membranes were then washed three times with TBST and then incubated, for 60 min at room temperature, with horseradish peroxidase-conjugated secondary antibodies (ref: 172-1011, Bio-Rad, Hercules, CA) diluted 1:10,000 in the blocking buffer. Following another three washes with TBST, immunoblots were developed using Western Lightning enhanced chemiluminescence Pro (PerkinElmer, Waltham, MA) and imaged using X-ray film or the LiCor Odyssey Fc system.

### RT-QuIC assay and buffer preparation

RT-QuIC reactions were performed in 384-well plates with a clear bottom (Nunc, NY). Recombinant α-synuclein (rPeptide, Athens, GA) was thawed from − 80 °C storage, reconstituted in HPLC-grade water (Sigma, St. Louis, MI) and filtered through a 100-kDa spin filter (Thermo Scientific) in 500-µl increments. All reagents used in the reaction buffers were purchased from Sigma. Then 10 μl of the biological sample (5 μg of total protein from the PBS-soluble fraction) was added to the wells containing 20 μl of the reaction buffer, 10 μl of 50 μM ThT and 10 μl of 0.5 mg/ml of monomeric recombinant α-synuclein. In every plate, positive (1 μg of α-synuclein preformed fibrils (rPeptide)) and negative (deonized water) controls were added. To ensure RT-QuIC reproducilility, a coefficient of variation no greater than 20% had been obtained when the positive control signals from different runs were compared. The plate was sealed and incubated at 37 °C in a BMG FLUOstar Omega plate reader with cycles of 1-min shaking (400 rpm double orbital) and 14-min rest. ThT fluorescence measurements (450 ± 10 nm excitation and 480 ± 10 nm emission, bottom read) were taken every 15 min for a period of 72 h.

### Conformational stability assays (CSA)

Twenty microliters of 2× guanidine hydrochloride (GdnCl) stocks were added to an equal volume of the PBS-soluble fraction of brain homogenates or RT-QuIC-derived α-synuclein fibrils to yield final GdnCl concentrations of 0, 1, 1.5, 2, 2.5, 3, 3.5 and 4 M, as previously described [24]. Briefly, PBS-soluble brain samples were incubated at room temperature with shaking for 120 min (800 rpm) before being diluted to 0.4 M GdnCl in PBS. Following high-speed ultracentrifugation at 100,000 *g* for 60 min at 4 °C, the pellets were resuspended in 1× LDS loading buffer and boiled for 10 min. Levels of residual α-synuclein were determined by SDS-PAGE followed by immunoblotting, as mentioned above. Densitometry was performed using ImageJ, and values were normalized to the sample with the highest intensity, which was set at 100%. GdnCl_50_ values, the concentration of GdnCl at which 50% of the aggregates are solubilized, were determined by nonlinear regression using the sigmoidal dose–response (variable slope) equation in GraphPad Prism (v9, San Diego, CA) with the top and bottom values fixed at 100 and 0, respectively.

### Thermolysin digestion

Thermolysin digestion was performed as previously described [8, 24], with minor modifications. A concentration of 50 μg/ml of thermolysin was added to the PBS-soluble brain homogenates. RT-QuIC-derived α-synuclein fibrils were diluted in 1 × PBS containing thermolysin at a concentration of 5 μg/ml. Samples were incubated at 37 °C with continuous shaking (600 rpm) for 60 min. Digestions were halted with the addition of EDTA to a final concentration of 2.5 mM, and samples were ultracentrifuged at 100,000 *g* for 60 min at 4 °C. Supernatant was discarded and pellets resuspended in 1× LDS buffer and analyzed by SDS-PAGE followed by immunoblotting, as described above.

### Electron microscopy

Aliquots (5 μl of α-synuclein fibril preparations) containing different RT-QuIC-derived α-synuclein fibrils were loaded onto freshly glow-discharged 400 mesh carbon-coated copper grids (Electron Microscopy Sciences, Hatfield, PA) and adsorbed for 1 min. Once dry, the grids were visualized using a Talos L120C transmission electron microscope (Thermo Fisher) using an acceleration voltage of 200 kV. Electron micrographs were recorded using an Eagle 4kx4k CETA CMOS camera (Thermo Fisher).

#### Statistics

Statistical analyses were performed using GraphPad Prism (v.9) with a significance threshold of *P* = 0.05. RT-QuIC relative fluorescence responses were also analyzed and plotted using the software GraphPad Prism (v.9). Comparisons were made using unpaired two-tailed *t*-tests or one-way ANOVA with Tukey’s multiple comparisons test. Two-tailed Spearman *r* non-parametric correlations were used to correlate different variables obtained from single individuals using SPSS (v.25, Chicago, IL).

## Results

### Screening 168 RT-QuIC conditions to detect α-synuclein seeding in MSA

**Fig. 1:**
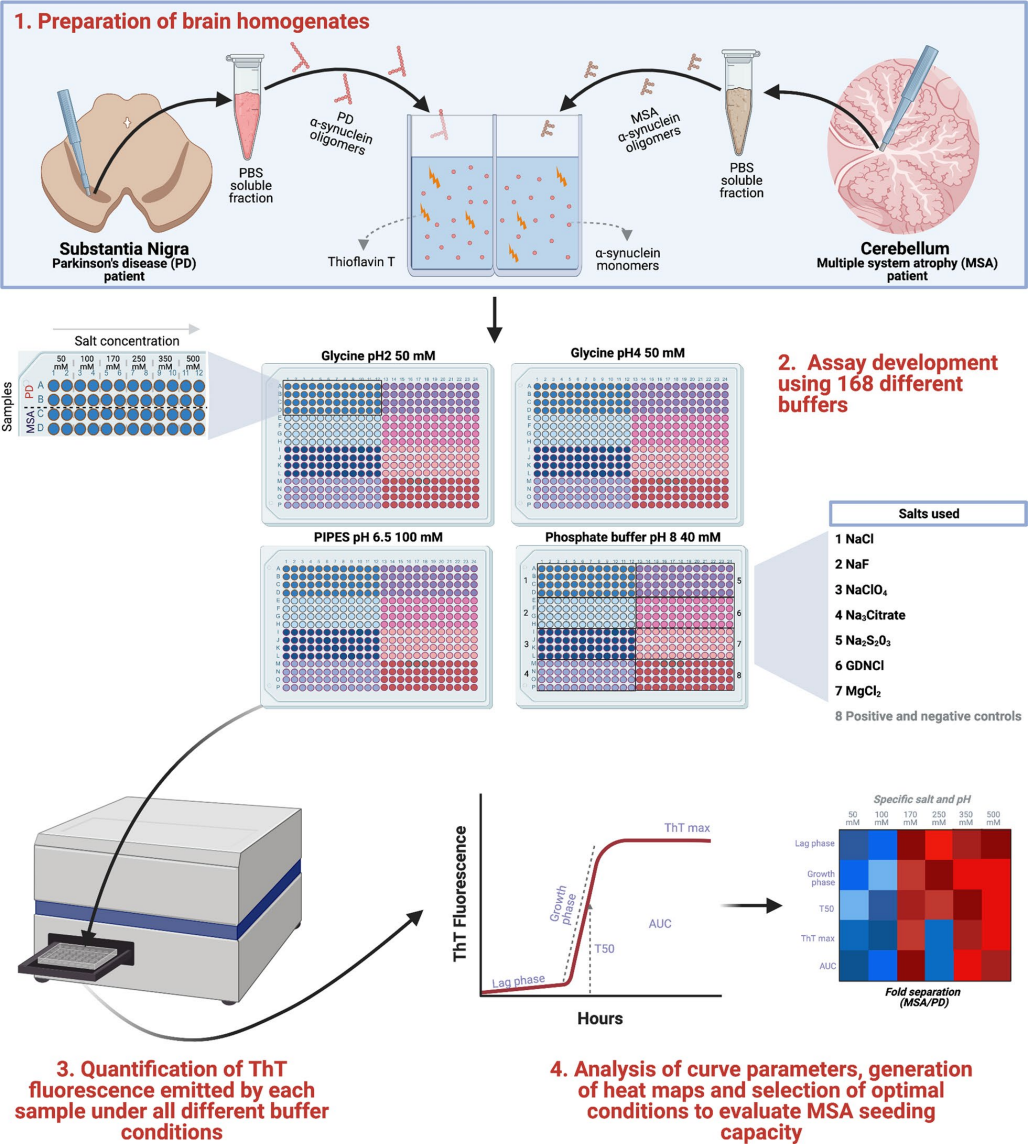
**Schematic of the RT-QuIC buffer discovery phase to evaluate α-synuclein seeding in MSA. **Step 1: Samples containing α-synuclein seeds were prepared from substantia nigra of PD or cerebellum of MSA patients. Step 2: The samples were incubated with 168 reaction buffers and subjected to cycles of shaking and resting at 37 °C. Step 3: ThT output was measured at multiple timepoints over 48 h. Step 4: Results were analyzed and heatmaps were generated to determine the optimal conditions to discriminate MSA-derived samples from PD-derived samples. Designed with Biorender.com

**Fig. 2:**
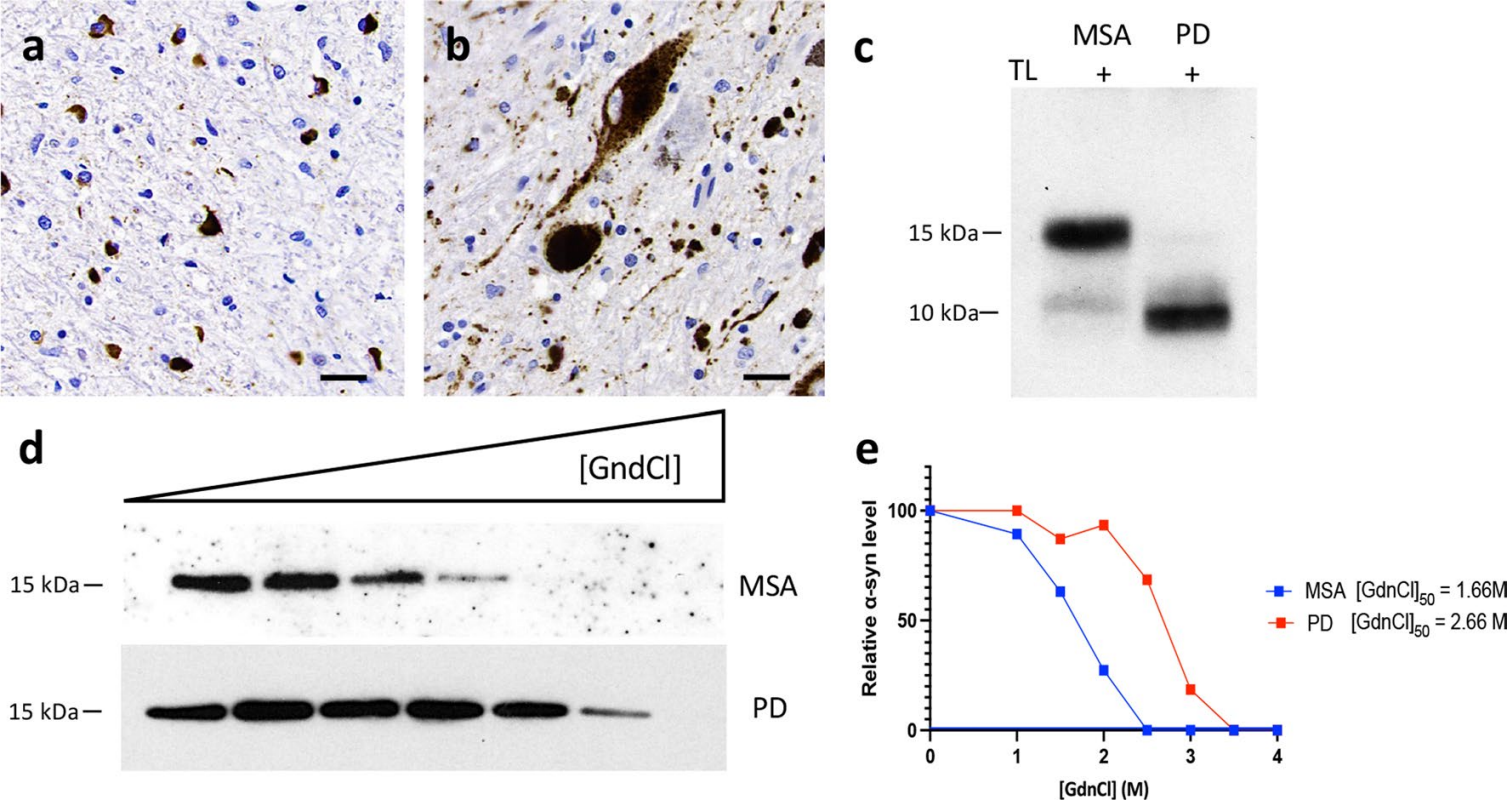
**Conformational discrimination of α-synuclein strains in PD and MSA brains.** **a** Representative immunohistochemistry image for aggregated α-synuclein in the cerebellum white matter from a MSA patient showing glial cytoplasmic inclusions. **b** Representative immunohistochemistry image for aggregated α-synuclein in the substantia nigra (SN) from a PD patient showing Lewy bodies and Lewy neurites. **c** Representative total α-synuclein (Syn-1 clone) immunoblots showing the thermolysin (TL) digestion of brain extracts from the cerebellum and the SN of a MSA and a PD patient, respectively. **d**, **e** Conformational stability assays for α-synuclein aggregates in brain extracts from patients with MSA or PD. Representative α-synuclein immunoblots (**d**) and the resultant denaturation curves **(e**) are shown. The curves depict mean residual α-synuclein values following treatment with the indicated concentrations of GdnCl. Higher GdnCl_50_ values were obtained for α-synuclein aggregates in the patient with PD than that in the patient with MSA

We hypothesized that changing the physicochemical factors that govern the in vitro amplification of amyloidogenic proteins would favor α-synuclein seeding in MSA. Based on the published success of using different ionic environments to enhance the sensitivity of different proteopathic seeding amplification assays [19], we conducted a systematic evaluation of 168 different reaction buffers, using an array of pH and salts, seeded with brain homogenates from one MSA and one PD patient (Fig. 1). We compared the α-synuclein RT-QuIC in the presence of strongly (Citrate^3−^ and S_2_O_3_^2−^), moderately (F^−^ and Cl^−^) and weakly (ClO_4_^−^) hydrated anions and cations (GdnCl and MgCl_2_) at high (500 and 350 mM), medium (250 and 170 mM) and low (100 and 50 mM) concentrations using 4 different pH (pH 2, pH 4, pH 6.5 and pH 8). In the resulting 168 buffers, equal amounts (5 μg) of total brain protein were used per reaction. A region rich in Lewy body pathology, the SN, from a PD patient and a region enriched with GCIs, the cerebellar white matter, from a MSA patient, were carefully microdissected using a 4-mm brain tissue punch and subsequently processed into crude homogenates, using PBS. In these two cases, conventional immunohistochemistry for disease-associated α-synuclein demonstrated abundant Lewy bodies and neurites in the SN of the PD patient and GCIs in the cerebellar white matter of the MSA patient (Fig. 2a, b). Next, we looked for further biochemical and structural differences in the α-synuclein brain homogenates. Preliminary studies were performed to compare the digestion of PBS-soluble α-synuclein with proteinase K or with thermolysin. We found that thermolysin digestion offered a superior discriminative cleavage pattern between MSA and PD brains, which was also replicated when the RT-QuIC products were digested. Therefore, all subsequent experiments report the results of thermolysin digestion. The band pattern of thermolysin-resistant α-synuclein species in MSA was different from that in PD. While both brain extracts possessed a band at ~ 10 kDa, the MSA extract displayed a prominent additional band at ~ 15 kDa (Fig. 2c). Finally, using CSA that measures the abilities of protein aggregate strains to resist denaturation by GdnCl, we found that α-synuclein aggregates from the MSA brain extract were significantly less stable than those present in the PD brain extract (Fig. 2d, e).

**Fig. 3:**
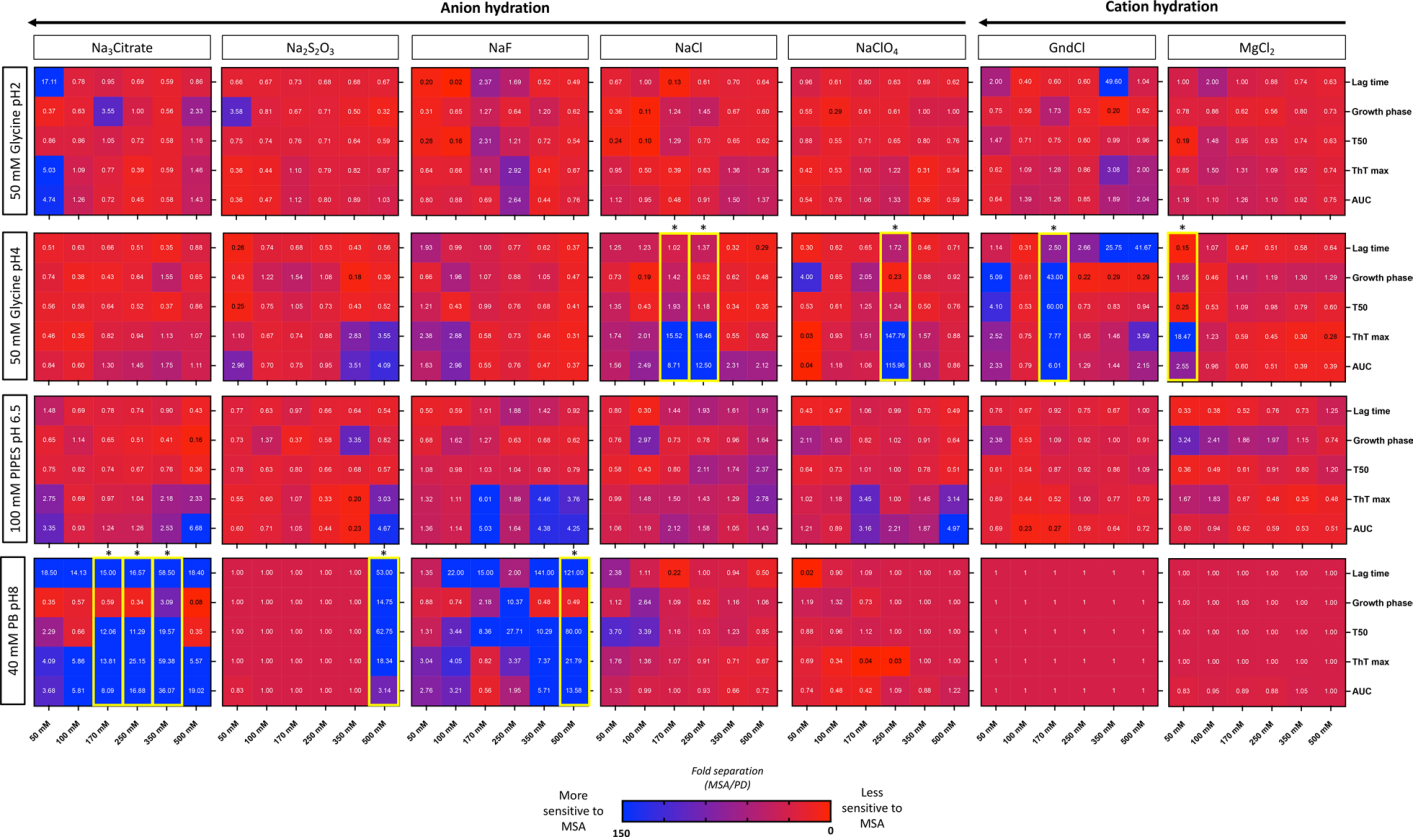
**Changes in RT-QuIC physicochemical factors promote the detection of α-synuclein seeding in MSA**. Fold separation of lag time, growth phase, T50, ThT max, and area under the curve (AUC) between reactions seeded with MSA- or PD-derived α-synuclein aggregates and amplified under 4 different pH using 7 salts at 6 concentrations. Larger numbers (blue) represent the conditions that showed a more favorable environment to detect MSA α-synuclein aggregation. When the fold separation is 1 (deep red), no discrimination between MSA and PD aggregation was found. Lower numbers (< 1, lighter red/purple) represent the conditions more favorable for detection of PD aggregation over MSA. *Buffers highlighted in yellow boxes represent the optimal conditions to detect α-synuclein seeding in MSA and discriminate MSA from PD

Following the characterization of the brain homogenates, to identify the optimal assay conditions that favor MSA seeding activity, we seeded 168 different RT-QuIC reaction buffers using 5 μg of MSA-derived or PD-derived PBS-soluble fraction. Four different RT-QuIC plates were ran in this phase (Fig. 1). Each condition was tested in quadruplicate and the same positive and negative controls were added in each plate. We then quantified 5 kinetic parameters from each reaction: lag time, growth phase, T50 (which corresponds to the time needed to reach 50% of maximum aggregation), ThT max or fluorescence peak, and area under the curve (AUC) of the fluorescence response. Each kinetic parameter was calculated as the mean of the values obtained from each quadruplicate. Importantly, we confirmed that no significant differences were observed in any of these 5 parameters between the positive and negative control samples run on all plates. Next, we calculated the fold separation of the 5 kinetic parameters examined by dividing the values obtained by the MSA and the PD brain homogenates, and generated heatmaps (Fig. 3). Then, to select the conditions that were most favorable to detect MSA-derived α-synuclein aggregation, we defined the AUC as the seeding parameter of interest as it incorporates all the kinetic features of each aggregation reaction, including the speed and extent of aggregation. Our analysis revealed that 19/168 buffers were able to detect predominantly PD-derived α-synuclein aggregation, showing an at least two-fold difference between PD and MSA curves; 109/168 buffers showed no major differences in the main parameters describing the kinetic curve of the RT-QuIC when the MSA aggregation pattern was compared to PD; while 40/168 buffers showed an at least two-fold difference between MSA-derived α-synuclein and PD-derived α-synuclein aggregation. From these 40 buffers, 10 conditions that displayed the highest differences were selected to further examine the parameters of kinetic curves (Additional file 1: Fig. S1). Interestingly, these buffers had a bimodal distribution, where higher differences between MSA-derived and PD-derived α-synuclein aggregation were observed with low-hydrated anions and cations when the reaction occurred at pH 4, while at pH 8 the highly hydrated anions had the best performance (Fig. 3). Based on the raw values obtained with each condition, we selected two buffers: buffer 1 (50 mM glycine, pH 4, 250 mM NaClO_4_) and buffer 2 (40 mM phosphate buffer [PB], pH 8, 350 mM Na_3_Citrate) for further analysis.

### Validation of the two optimal conditions for assessing α-synuclein seeding in MSA

**Fig. 4:**
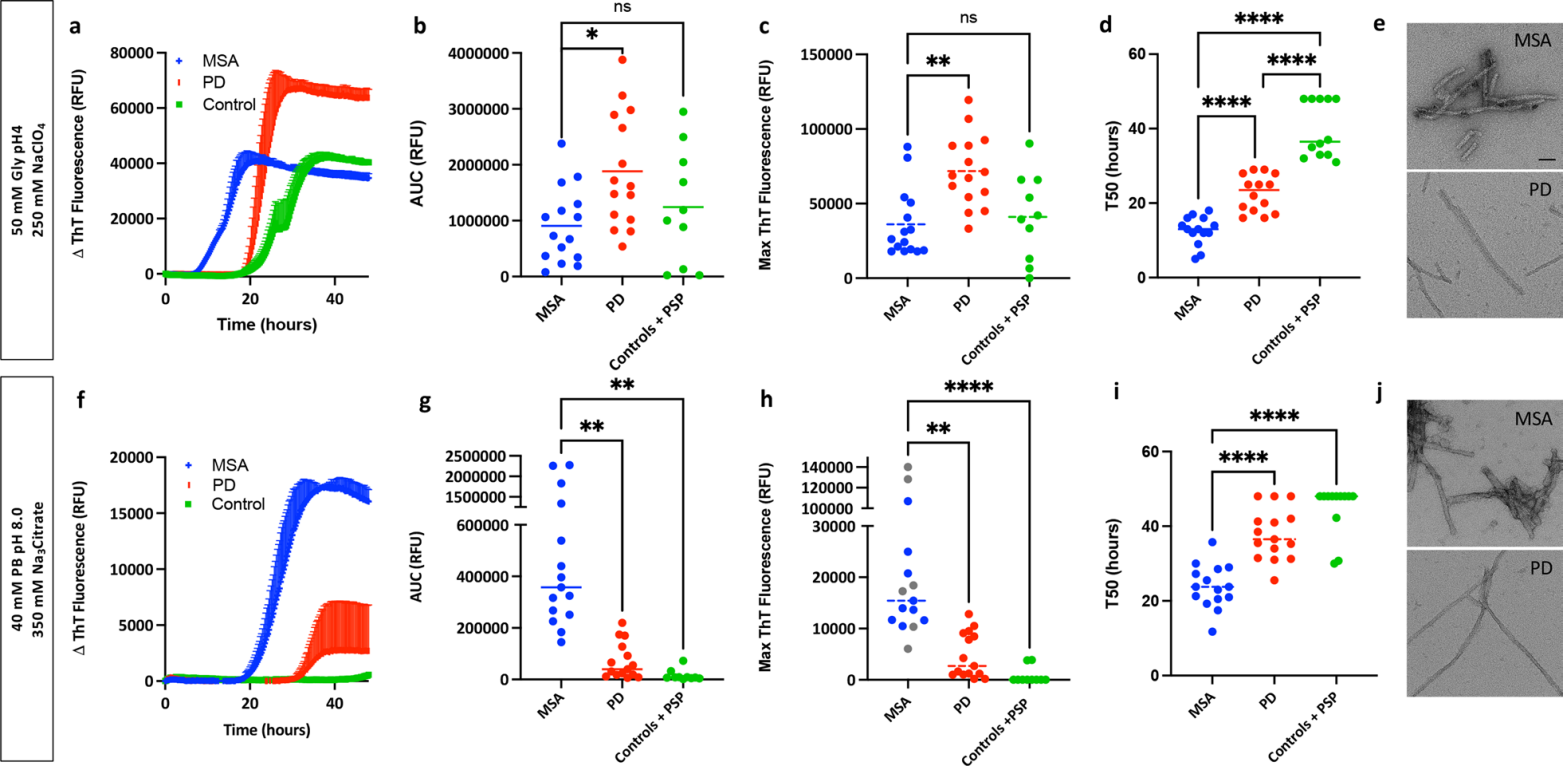
**Interaction of ThT dye with α-synuclein aggregates derived from patients with PD or MSA is dependent on the RT-QuIC reaction buffer used.** **a** Aggregation curves of α-synuclein in the presence of brain homogenates from a representative PD patient, a representative MSA patient, and a control subject, amplified with buffer 1 (50 mM Gly, pH 4, 250 mM NaClO_4_). Data are mean ± SEM of representative subjects measured in quadruplicate. **b**–**d** Area under the curve values (**b**), maximum ThT fluorescence values (**c**), and T50 (**d**) for PD (*n* = 15), MSA (*n* = 15), supranuclear progressive palsy (*n* = 5) and controls (*n* = 5). Each dot represents an individual biological sample measured in quadruplicate. **e** At the ultrastructural level, the MSA-derived RT-QuIC fibrils were thicker and more twisted than the PD-derived fibrils, as determined by electron microscopy. **f**–**i** Same as that for **a**–**d**, except that aggregation was performed in buffer 2 **(**40 mM PB, pH 8, 350 mM Na_3_Citrate**). j** At the ultrastructural level, the MSA-derived RT-QuIC fibrils were thicker and more twisted than the PD-derived fibrils, as determined by electron microscopy. *P < 0.05, **P < 0.01, ***P < 0.001, ****P < 0.0001 by one-way analysis of variance (ANOVA) followed by Tukey’s multiple comparison test. Scale bar, 25 nm

The two optimal buffers were further validated in a larger cohort of disease samples for their performace in detecting MSA-derived α-synuclein seeding activity and differentiating it from PD-derived α-synuclein seeding activity. We microdissected the SN of 15 subjects with MSA, 15 subjects with PD, 5 subjects with supranuclear progressive palsy and 5 controls and processed the samples into PBS-soluble homogenates. All subjects had a detailed neuropathological examination (Additional file 1: Table S1). To exclude the potential differences in α-synuclein aggregate recovery between MSA and PD subjects during the protein extraction, we quantified the amount of total and aggregated α-synuclein using ELISA. No differences either in the overall amount of total α-synuclein or in the aggregated α-synuclein were found between the two groups of patients (Additional file 1: Fig. S2). Equal amounts (5 μg) of total protein from each of the 40 subjects were used to seed the reactions, using the two buffer conditions, with all samples run in quadruplicate on the same plate. To illustrate the typical profile of α-synuclein RT-QuIC aggregation for samples of PD and MSA, we plotted data from one representative MSA, one PD and one control case amplified under the buffer 1 condition (Fig. 4a). The maximum fluorescence and the AUC were consistently different between PD and MSA, with samples from patients with MSA consistently aggregating faster while always reaching a lower fluorescence plateau than samples from patients with PD (Fig. 4b, c). When amplified under the buffer 2 condition, for the same cases we observed that samples from patients with MSA still aggregated faster but in contrast, they reached a higher fluorescence plateau than those from patients with PD (Fig. 4f). Furthermore, significant differences in the maximum fluorescence and the AUC were observed between PD and MSA patients (Fig. 4g, h). To test whether the amount of ThT bound to the MSA and PD fibrils depends on the physicochemical factors of the buffer, we investigated if the end products from the RT-QuIC assay amplified under the two conditions showed structural differences when examined using electron microscopy (Fig. 4e, j). Interestingly, despite the striking differences in the ThT kinetics between the two buffers, under both conditions the fibrils derived from the PD samples consistently showed a thin and straight morphology, while the fibrils derived from the MSA samples were consistently thicker and twisted. However, we were not able to determine whether the PD or the MSA fibrils generated under different conditions presented structural differences. In addition, the MSA samples did not show significant differences in either the maximum fluorescence or in the AUC compared to controls or supranuclear progressive palsy subjects under the buffer 1 condition (Fig. 4b, c). However, the MSA samples showed faster aggregation (T50) than the control and supranuclear progressive palsy group (Fig. 4d). When the buffer 2 condition was examined, we found an optimal distinction between MSA and PD and maximized the kinetic separation from controls and supranuclear progressive palsy subjects in the maximum fluorescence, T50 and the AUC (Fig. 4g–i). Therefore, the buffer 2 (40 mM PB, pH 8, 350 mM Na_3_Citrate) condition was selected as the optimal working buffer to study MSA α-synuclein seeding in RT-QuIC assays.

### Characterization of the RT-QuIC end-products

**Fig. 5:**
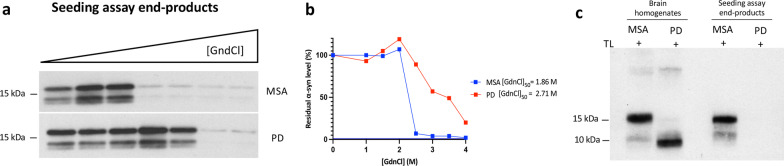
**RT-QuIC reaction products seeded from MSA α-synuclein fibrils are conformationally distinguishable from RT-QuIC reaction products seeded from PD-derived fibrils.** **a**, **b** Conformational stability assays for the RT-QuIC-derived MSA and PD fibrils. Representative α-synuclein immunoblots (**a**) and the resultant denaturation curves (**b**) are shown. MSA-derived fibrils are less stable than PD-derived fibrils in the epitope (amino acids 15–123) used to probe the structure. **c** Immunoblots of PBS-soluble α-synuclein species in brain homogenates from PD and MSA patients and their RT-QuIC-derived fibrils with thermolysin (TL) digestion. TL-resistant α-syn species were present in MSA and PD brain extracts, but TL-resistant α-syn species were only detectable in the MSA RT-QuIC-derived fibrils

To gain further insight into the structures of aggregates amplified from patients with PD or MSA, we analyzed the buffer 2 RT-QuIC end-products by CSA and thermolysin digestion. For these experiments, we examined the RT-QuIC end-products derived from the PD and MSA subjects at the screening phase. As previously mentioned, under the CSA, the aggregates present in the MSA brain homogenate were significantly less stable following exposure to GdnCl than those found in the PD brain homogenate (Fig. 2d, e). This difference in conformational stability was preserved in the epitope (amino acids 15–123) used to probe the structure following the *in-vitro* amplification by RT-QuIC, and the end-products derived from the MSA brain were less stable than the PD ones (Fig. 5a, b). Results of thermolysin digestion showed that the MSA homogenate and the MSA-derived RT-QuIC end-products possessed a band at ~ 10 kDa and a prominent additional band at ~ 15 kDa (Fig. 5c). However, while the PD homogenate showed a band at ~ 10 kDa, the α-synuclein present in the PD RT-QuIC end-product was not thermolysin-resistant. These results, together with the ultrastructural data, show that under our assay conditions, the RT-QuIC reaction products seeded from MSA α-synuclein fibrils are conformationally distinguishable from RT-QuIC reaction products seeded from PD-derived fibrils.

### MSA patients present distinct α-synuclein seeding

**Fig. 6:**
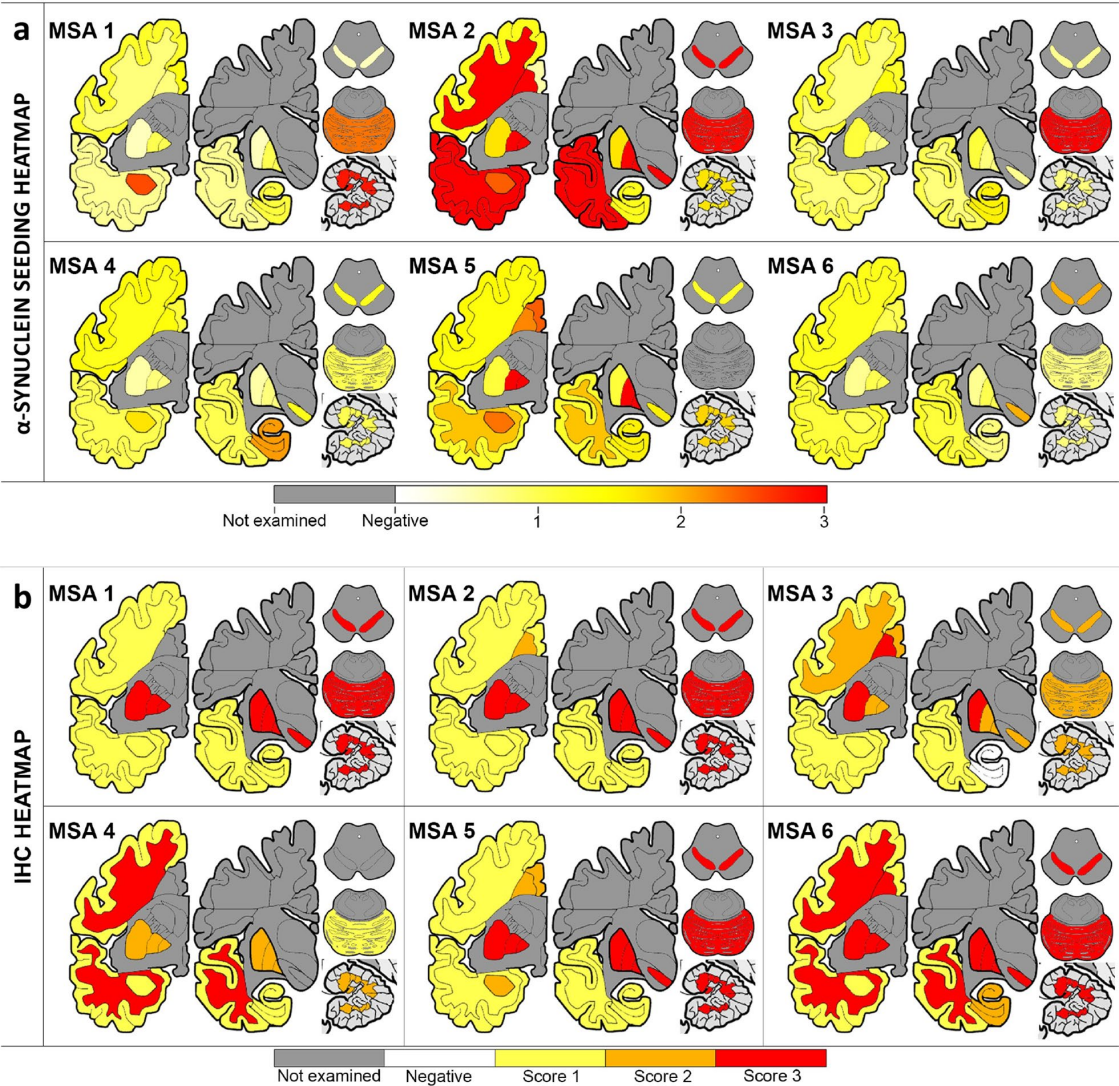
**Extensive heterogeneity of α-synuclein seeding activity across different MSA patients and different brain regions.** Heat mapping of α-synuclein seeding (**a**) assessed by RT-QuIC and of aggregated α-synuclein (**b**) evaluated by immunohistochemistry using the conformational α-synuclein 5G4 antibody. The α-synuclein seeding ranged from white (none) through yellow (low) and orange (medium) to red (high). The semiquantitative score of the severity of α-synuclein pathology ranged from white (none) through yellow (mild) and orange (moderate) to red (severe). Grey colored cortical regions indicate that the region was not evaluated

In addition to the observed differences in the AUC and ThT max values among the different groups of patients examined, we observed up to ten-fold differences in AUC and ThT max values among the 15 MSA subjects (Fig. 4g, h). Based on the data derived from the SN samples, the subjects with MSA included in our study were further subtyped into 3 groups: high, intermediate and low seeders, according to their ability to misfold monomers of recombinant α-synuclein in vitro. To test the hypothesis that heterogeneity in α-synuclein seeding exists in MSA, we selected six from the 15 MSA subjects (Fig. 4h), and classified them as low seeders (subjects MSA 1 and MSA 6), intermediate seeders (subjects MSA 3 and MSA 4) and high seeders (subjects MSA 2 and MSA 5) according to their α-synuclein seeding behavior. To assess whether these distinctions could be due to differences in seed concentration or seed characteristics, we performed end-point dilution of the cerebellar PBS-soluble fraction from two MSA cases classified as high and low seeders, respectively (Additional file 1: Fig. S3). Regardless of the amount of brain homogenate used to seed the RT-QuIC reaction, the seeding activity of the misfolded α-synuclein from the high-seeder case was more potent than that from the low-seeder case (Additional file 1: Fig. S3). Then, the α-synuclein seeding behavior was assessed in 13 different brain regions from these subjects. From each case, we micro-dissected and subsequently prepared a PBS-soluble extract from the following regions: anterior cingulate cortex, anterior cingulate white matter, frontal cortex, frontal white matter, putamen, globus pallidus, amygdala, hippocampus, temporal cortex, temporal white matter, SN, pons base and cerebellar white matter (Additional file 1: Fig. S4). All samples were run in quadruplicate, and the RT-QuIC curve obtained by each reaction was evaluated as a measure of α-synuclein seeding behavior (Additional file 1: Fig. S5). The AUC values were converted into scores ranging from 0 to 3. With these values, we generated a heatmap of α-synuclein seeding to illustrate the differences between brain regions and patients (Fig. 6a). Interestingly, based on the mean AUC values across the regions, the two high-seeder patients had significantly higher AUC values than the intermediate- (*P* < 0.0001) or low-seeder (*P* < 0.0001) patients. When the different brain regions were examined individually, the pons base showed the highest α-synuclein seeding. Surprisingly, the cerebellar white matter and the putamen showed the lowest α-synuclein seeding. When the different cortices were examined, we found that the frontal and temporal cortex displayed medium seeding, while the cingulate cortex had higher seeding. A higher seeding was consistently observed in the white matter of the three cortices when compared to the grey matter. Overall, the hippocampus displayed medium seeding, while the α-synuclein present in globus pallidus, SN and the amygdala had higher seeding.

### Biochemical and neuropathological characterization of α-synuclein

The factors that underlie the ability of α-synuclein from different patients with MSA to drive higher *versus* lower seeding are presently unknown. To investigate this further, we conducted a biochemical examination of α-synuclein derived from the brain homogenates coupled with a detailed immunohistochemical mapping of α-synuclein deposition in corresponding samples. First, we quantified the amount of total and aggregated α-synuclein using ELISA and correlated these measures with their α-synuclein seeding behavior. The MSA cases had 0.6–4 ng of total α-synuclein per mg of tissue in the PBS-soluble fraction. No differences in the overall amount of α-synuclein were found among high, low and intermediate seeders. The regions with more abundant total α-synuclein were amygdala and the frontal cortex, whereas the cerebellar white matter and the pons had less amount of α-synuclein (Additional file 1: Fig. S6). The amount of aggregated α-synuclein was quantified using the α-Synuclein Patho ELISA. MSA cases had 3–362 pg of aggregated α-synuclein per mg of tissue in the PBS-soluble fraction. Interestingly, the amount of aggregated α-synuclein was significantly higher (*P* = 0.0165) in the low seeders compared to the high seeders. The regions that had a higher burden of aggregated α-synuclein were the temporal and the anterior cingulate cortex, whereas the cerebellar white matter and the pons had the least aggregated α-synuclein (Additional file 1: Fig. S6). The total amount of α-synuclein in the PBS-soluble fraction did not correlate with either the amount of aggregated α-synuclein (*P* = 0.171) or the α-synuclein seeding activity (*P* = 0.648). However, the levels of aggregated α-synuclein were negatively correlated with the α-synuclein seeding activity (*P* = 0.035, *r* =  − 0.241). These data, together with the striking seeding activity in soluble fractions from brain regions without noticeable accumulations of α-synuclein, led us to test whether the heterogeneity among regions and patients was also maintained if larger, sarkosyl-insoluble (SI), α-synuclein aggregates were used to seed the RT-QuIC assay. To test this hypothesis, we extracted α-synuclein SI aggregates from the cerebellum, putamen and frontal cortex from one MSA case classified as a low seeder (MSA 1) and one MSA case categorized as a high seeder (MSA 2). Equal amounts (5 μg) of total protein were used to seed all the reactions, and quadruplicates from these samples together with their corresponding PBS-soluble fractions were evaluated. Furthermore, owing to the insoluble nature of the SI aggregates and possible inaccessibility of the fibril ends, we also included sonicated SI samples. When the RT-QuIC curves were evaluated, there was no significant difference between regions for the SI aggregates in either subject. However, the SI aggregates from the three brain regions of the high seeder (MSA 2) aggregated faster and reached a higher fluorescence plateau than the SI aggregates from the low seeder (MSA 1, Additional file 1: Fig. S7a). Regardless of the region or the patient examined, the SI aggregates promoted faster aggregation and reached a higher fluorescence plateau than the α-synuclein aggregates present in the corresponding PBS-soluble fraction (Additional file 1: Fig. S7b, c; Additional file 1: Fig. S8). The same pattern was observed when the differences in the α-synuclein aggregation kinetics were examined between SI aggregates with and without sonication, where the sonicated SI aggregates promoted a faster aggregation and reached a higher fluorescence plateau than the non-sonicated (Additional file 1: Fig. S7b, c).

Finally, we aimed to evaluate whether the heterogeneity of α-synuclein seeding was reflected in immunohistological findings in the brains of the same subjects. First, we performed epitope mapping to compare which α-synuclein antibody was able to detect the most MSA α-synuclein pathology in formalin-fixed paraffin-embedded tissue sections. Using consecutive sections from the putamen and the cerebellum from our cohort of 6 MSA patients, we performed morphometric analysis using four different α-synuclein antibodies: the 5G4 conformational antibody that recognizes aggregated forms of α-synuclein, the pSyn#64 that recognizes Ser129 phosphorylated α-synuclein, and two antibodies against the truncated (C-terminal cleaved) and nitrated forms of α-synuclein (clones A15127A and Syn514, respectively) (Additional file 1: Fig. S9). The conformational 5G4 antibody consistently exhibited more α-synuclein pathology in all the cases and regions examined, followed by the two antibodies that recognize Ser129 phosphorylated α-synuclein, and truncated α-synuclein (Additional file 1: Fig. S9m). Thus, semi-quantitative analysis of the α-synuclein aggregation burden was performed using the 5G4 antibody (Fig. 6b and Additional file 1: Fig. S10). The cerebellum and the putamen showed the highest aggregated α-synuclein, with the SN, the pons base and the globus pallidus also exhibiting a high burden of aggregated α-synuclein. The grey matter from the temporal and frontal cortex had less amount of aggregated α-synuclein. In addition to the semi-quantitative analysis of the total burden of aggregated α-synuclein, the burden of GCIs and NCIs was also examined (Additional file 1: Fig. S11). As expected, the presence of NCIs was less frequent than the presence of GCIs. The pons base, SN and putamen, followed by the hippocampus and the anterior cingulate cortex, were the regions where more NCIs were evident. When the GCIs burden was evaluated, the putamen and cerebellum, followed by the globus pallidus and the SN were the regions with more abundant GCIs, while the hippocampus and the frontal cortex has less abundant GCIs. Surprisingly, there was no significant difference in the burden of GCIs, NCIs or aggregated α-synuclein between the high, low and intermediate seeders, and no correlation was found between the burden of GCIs (*P* = 0.957) or NCIs (*P* = 0.252) with the α-synuclein seeding.

## Discussion

In this study, we showed that the physicochemical factors that govern the in vitro amplification of α-synuclein can be tailored to generate strain-specific reaction buffers for use in RT-QuIC. Using this novel approach, we have generated a streamlined RT-QuIC assay that (1) is able to measure the α-synuclein seeding of 96 MSA samples, run in quadruplicate, in less than 48 h; (2) requires the use of a minimal amount of commercially available recombinant α-synuclein monomer (5 μg) per well; (3) requires the use of a minimal amount of brain material (5 μg); (4) generates RT-QuIC-derived α-synuclein fibrils that are conformationally distinct between patients with different synucleinopathies; and (5) is capable of subtyping MSA brains according to their α-synuclein seeding behavior.

MSA is clinically and pathologically heterogeneous, and although the distribution of GCIs and NCIs might correlate with the predominant clinical features [25, 26], the burden of α-synuclein inclusions does not fully explain the differences in clinical presentation and rate of disease progression exhibited in MSA. In recent years, there is mounting evidence highlighting the important role of amyloidogenic proteins in soluble brain fractions in seeding pathologic aggregation in a prion-like manner, which might contribute to the clinical heterogeneity seen in patients with neurodegenerative diseases [21]. The capacity of misfolded α-synuclein to template monomeric α-synuclein has been exploited by several groups to generate an RT-QuIC assay able to measure the seeding kinetics of α-synuclein in a range of PD samples [14, 17, 27–29]. However, the reliable detection of α-synuclein seeding in MSA-derived samples has been restricted to a couple of studies [18, 30]. To identify the optimal conditions to evaluate α-synuclein seeding in MSA-derived samples, we performed a comprehensive analysis of 168 different reaction buffers, covering an array of pH and salts. We then validated two conditions that conferred the optimal ability to discriminate between PD- and MSA-derived samples in a larger cohort of neuropathologically confirmed cases. Although same samples were used to seed the RT-QuIC reactions, the two different buffers resulted in significantly divergent kinetics of the curves, suggesting that the accessibility or the interaction of ThT with α-synuclein from MSA and PD can be modulated by the type of salt, pH value or ionic strength of the reaction buffer [18, 19, 31]. We selected buffer 2 (40 mM PB, pH 8, 350 mM Na_3_Citrate) as the optimal condition with which to evaluate α-synuclein seeding in MSA, and further demonstrated that the RT-QuIC-derived MSA-fibrils maintained the biochemical properties of the MSA aggregates used to seed the reaction.

Our multi-region mapping of α-synuclein seeding across 13 different brain regions in MSA revealed that the pons base had the highest seeding. Surprisingly, α-synuclein extracted from two of the most-affected regions in MSA, the cerebellum and the putamen, exhibited the lowest seeding. A possible explanation is that the analysis of the α-synuclein seeding was performed using the PBS-soluble fraction. It is plausible that the seeding activity of α-synuclein could diminish over time as the more soluble seeding-competent α-synuclein species, present at earlier stages of disease, become sequestered in larger insoluble aggregates at later stages of the disease. This could then result in reduced seeding of α-synuclein extracted from regions affected earlier in the disease course [21, 32]. This possibility led us to compare the seeding behavior of SI α-synuclein aggregates and the α-synuclein seeds present in the PBS-soluble fraction in different brain regions. When the α-synuclein seeding was compared, regional differences were only observed for the PBS-soluble fraction, but not for the SI aggregates. However, regardless of the region or the patient examined, the SI aggregates consistently promoted faster aggregation than the α-synuclein seeds in the PBS-soluble fraction, suggesting that there are more competent α-synuclein species in the SI fraction. Our findings support earlier observations that modifications and solubility of α-synuclein in MSA may be more widespread than histopathology [33, 34] and might be different between distinct synucleinopathies [34]. Future research should investigate the curious finding that PBS-soluble α-synuclein is the major contributor to the observed regional differences in the seeding behavior in MSA. Interestingly, and irrespective of the differences found between the SI and the soluble fraction, striking differences in the seeding activity between patients were also observed.

Our study is subject to some limitations. Our data clearly demonstrate that the selection of the right conditions to conduct the amplification of the seeds is of major relevance. Collectively, in this field, our understanding of the optimal in vitro physicochemical factors with which specific seed conformers can propagate is still in its infancy. Using suboptimal conditions, the absence of seeding activity could reflect the absence of seeds, but might also reflect an inability of the specific RT-QuIC conditions to support the amplification of those particular seeds [35].

Our findings pave the way for future studies to address the molecular mechanisms underlying the ability of α-synuclein from different MSA patients to drive higher *versus* lower seeding. Indeed, our results support a model whereby the heterogeneity observed both between different brain regions and between different MSA patients might be due to the differences in the cellular environment [6, 8] and to the presence of posttranslational modifications [9] and other cofactors, such as p25α [36], that may confer a selective pressure for one α-synuclein conformation over another [6].

## Conclusion

This study demonstrated that the physicochemical factors that govern the in vitro amplification of α-synuclein can be tailored to generate strain-specific reaction buffers for use in RT-QuIC. Using this novel approach, we have found unexpected differences in seed-competent α-synuclein across a cohort of neuropathologically comparable MSA brains. Understanding the relationship between the seeding differences and biological activity of α-synuclein will be critical for future subclassification of MSA, which should go beyond the conventional clinical and neuropathological phenotyping and consider the structural and biochemical heterogeneity of α-synuclein present in these patients. This will allow the development of new personalized therapeutic strategies for this devastating disease. Furthermore, a deeper understanding of how physicochemical factors influence the aggregation of different α-synuclein strains will provide support for the development of vitally needed, rapid and sensitive in vivo assays for the diagnosis of MSA and other synucleinopathies.

## Supplementary Information

**Additional file 1.** **Fig. S1.** The differential interaction of ThT with α-synuclein aggregates derived from patients with PD or MSA depends on the RT-QuIC reaction buffer. **Fig. S2.** The levels of total and aggregated α-synuclein in the Substantia Nigra are relatively uniform between the MSA and the LBD patients included in the validation phase. **Fig. S3.** End-point dilution alpha-synuclein RT-QuIC analysis of the cerebellar PBS-soluble fraction from two MSA cases classified as high and low seeders. **Fig. S4.** Dissection of brain regions for protein extraction and α-synuclein seeding evaluation. **Fig. S5.** The inter-individual α-synuclein seeding and intra-individual α-synuclein seeding behavior is distinct between MSA patients and brain regions. **Fig. S6.** The levels of total α-synuclein in each brain region are relatively uniform but the burden of aggregated α-synuclein varies across patients and brain regions in MSA patients. **Fig. S7.** The inter-individual but not the intra-individual α-synuclein seeding heterogeneity is preserved using the sarkosyl insoluble fraction. **Fig. S8.** The SI fraction promotes a faster aggregation and reaches a higher fluorescence plateau than the PBS-soluble fraction. **Fig. S9.** The extent of pathology detected by different α-synuclein antibodies is not uniform. **Fig. S10.** GCIs and NCIs deposition across different brain regions in MSA patients. **Fig. S11.** The burden of GCIs and NCIs varies across different brain regions in MSA. **Table S1.** Demographic and neuropathological diagnosis of the subjects included in this study.

## Data Availability

The data used and/or analysed during the current study are available from the corresponding author on reasonable request.

## Abbreviations

CSA: Conformational stability assay
CSF: Cerebrospinal fluid
GCI: Oligodendrocytic cytoplasmic inclusion
MSA: Multiple system atrophy
PD: Parkinson’s disease
PMCA: Protein misfolding cyclic amplification
RT-QuIC: Real-Time Quaking-induced Conversion
ThT: Thioflavin T
