# Supplementary material for: Alpha-synuclein seeding shows a wide heterogeneity in multiple system atrophy
Source: Transl Neurodegener. 2022 Feb 7;11:7. doi: 10.1186/s40035-022-00283-4 (PMC8819887; doi:10.1186/s40035-022-00283-4)
Supplement: Supplementary file 1 — Additional file 1. Fig. S1. The differential interaction of ThT with α-synuclein aggregates derived from patients with PD or MSA depends on the RT-QuIC reaction buffer. Fig. S2. The levels of total and aggregated α-synuclein in the Substantia Nigra are relatively uniform between the MSA and the LBD patients included in the validation phase. Fig. S3. End-point dilution alpha-synuclein RT-QuIC analysis of the cerebellar PBS-soluble fraction from two MSA cases classified as high and low seeders. Fig. S4. Dissection of brain regions for protein extraction and α-synuclein seeding evaluation. Fig. S5. The inter-individual α-synuclein seeding and intra-individual α-synuclein seeding behavior is distinct between MSA patients and brain regions. Fig. S6. The levels of total α-synuclein in each brain region are relatively uniform but the burden of aggregated α-synuclein varies across patients and brain regions in MSA patients. Fig. S7. The inter-individual but not the intra-individual α-synuclein seeding heterogeneity is preserved using the sarkosyl insoluble fraction. Fig. S8. The SI fraction promotes a faster aggregation and reaches a higher fluorescence plateau than the PBS-soluble fraction. Fig. S9. The extent of pathology detected by different α-synuclein antibodies is not uniform. Fig. S10. GCIs and NCIs deposition across different brain regions in MSA patients. Fig. S11. The burden of GCIs and NCIs varies across different brain regions in MSA. Table S1. Demographic and neuropathological diagnosis of the subjects included in this study. [file 40035_2022_283_MOESM1_ESM.docx]

**Supplementary material**

**
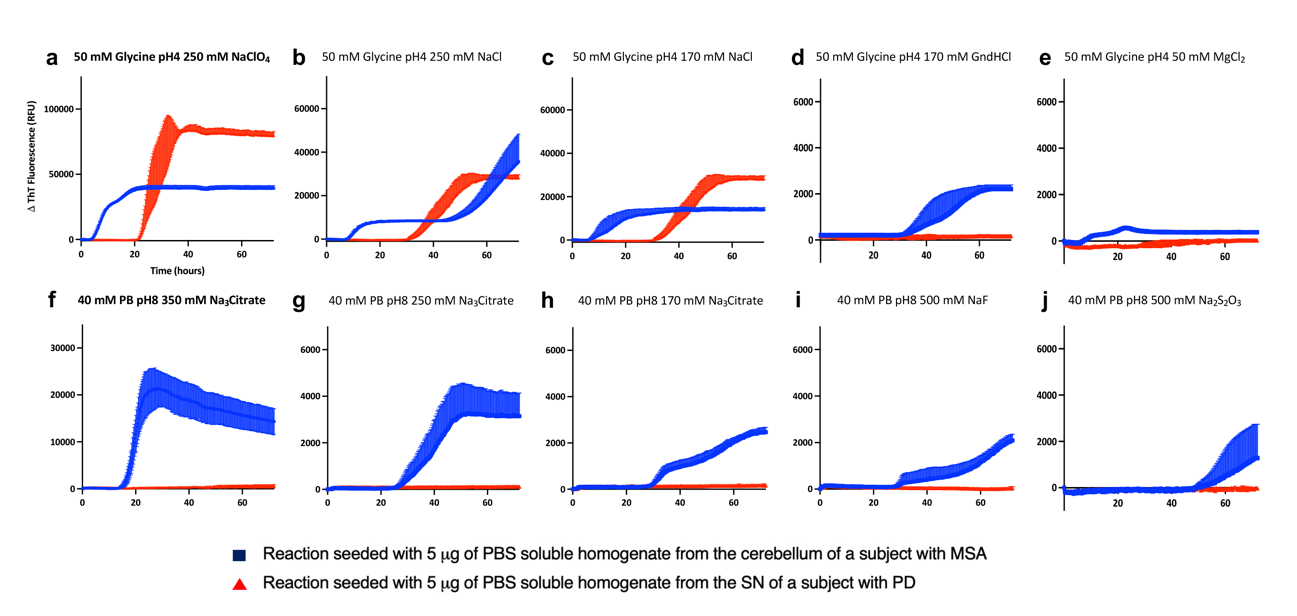
**

**Figure S1: Differential interaction of ThT with α-synuclein aggregates derived from patients with PD or MSA depends on the RT-QuIC reaction buffer.** Kinetic curves showing α-synuclein seeding activity from reactions seeded with 5 μG of total protein from the cerebellum of a MSA patient (blue curves) and the SN of a PD patient (red curves). The same samples were amplified using **a)** 50 mM glycine pH 4 250 mM NaClO_4_ **b)** 50 mM glycine pH 4 250 mM NaCl **c)** 50 mM glycine pH 4 170 mM NaCl **d)** 50 mM glycine pH 4 170 mM GndCl **e)** 50 mM glycine pH 4 50 mM MgCl_2_ **f)** 40 mM PB pH 8 350 mM Na_3_Citrate **g)** 40 mM PB pH 8 250 mM Na_3_Citrate **h)** 40 mM PB pH 8 170 mM Na_3_Citrate **i)** 40 mM PB pH 8 500 mM Na_3_Citrate and **j)** 40 mM PB pH 8 500 mM Na_2_S_2_O_3_ as RT-QuIC reaction buffers. Each curve depicts the quadruplicate mean ± s.e.m.

**Figure S2: The levels of total and aggregated** α**-synuclein in the Substantia Nigra are relatively uniform between the MSA and the LBD patients included in the validation phase.** The amount of total α-synuclein was quantified, using an ELISA assay, in the Substantia Nigra of **a)** 15 MSA and 15 LBD subjects **b)** no significant differences were found between the two groups of patients. The amount of aggregated α-synuclein was quantified using the α-synuclein Patho ELISA assay (that uses the 5G4 antibody as capture antibody) **c)** in the same region and patients and **d)** no significant differences were found between the two groups of patients.

**
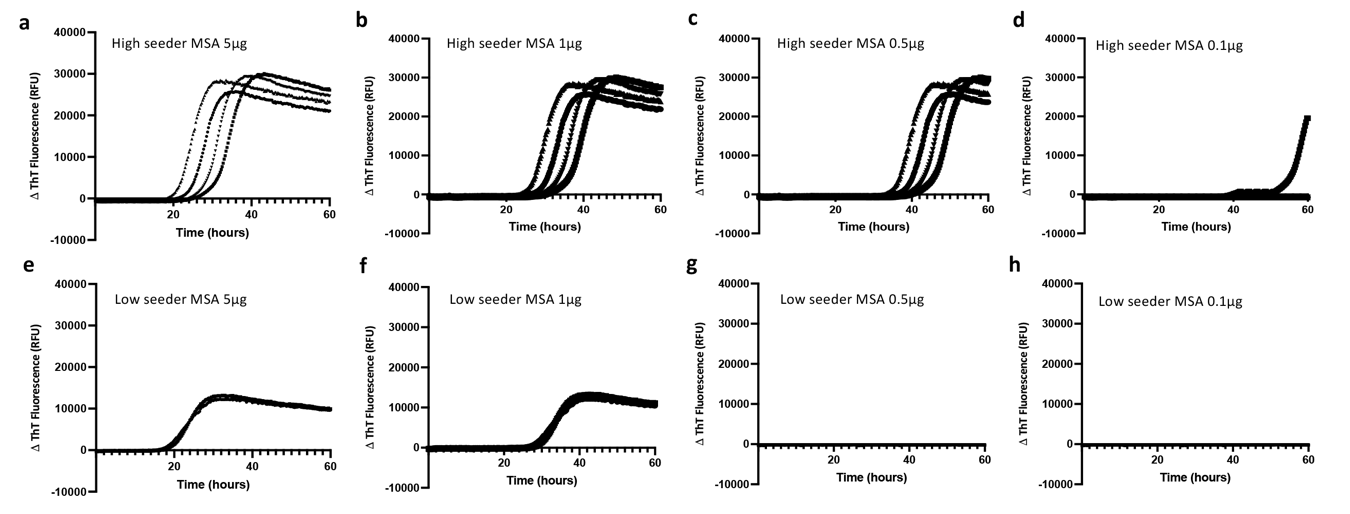
**

**Figure S3: End-point dilution alpha-synuclein RT-QuIC analysis of the cerebellar PBS-soluble fraction from two MSA cases classified as high and low seeders.** Kinetic curves of α-synuclein seeding activity measured by RT-QuIC of **a)** 5 μg, **b)** 1 μg, **c)** 0.5 μg and **d)** 0.1 μg of the PBS-soluble fraction and of **e)** 5 μg, **f)** 1 μg, **g)** 0.5 μg and **h)** 0.1 μg of the PBS-soluble fraction from the cerebellum of two MSA subjects. Each curve depicts each of the four replicates made per condition.

**
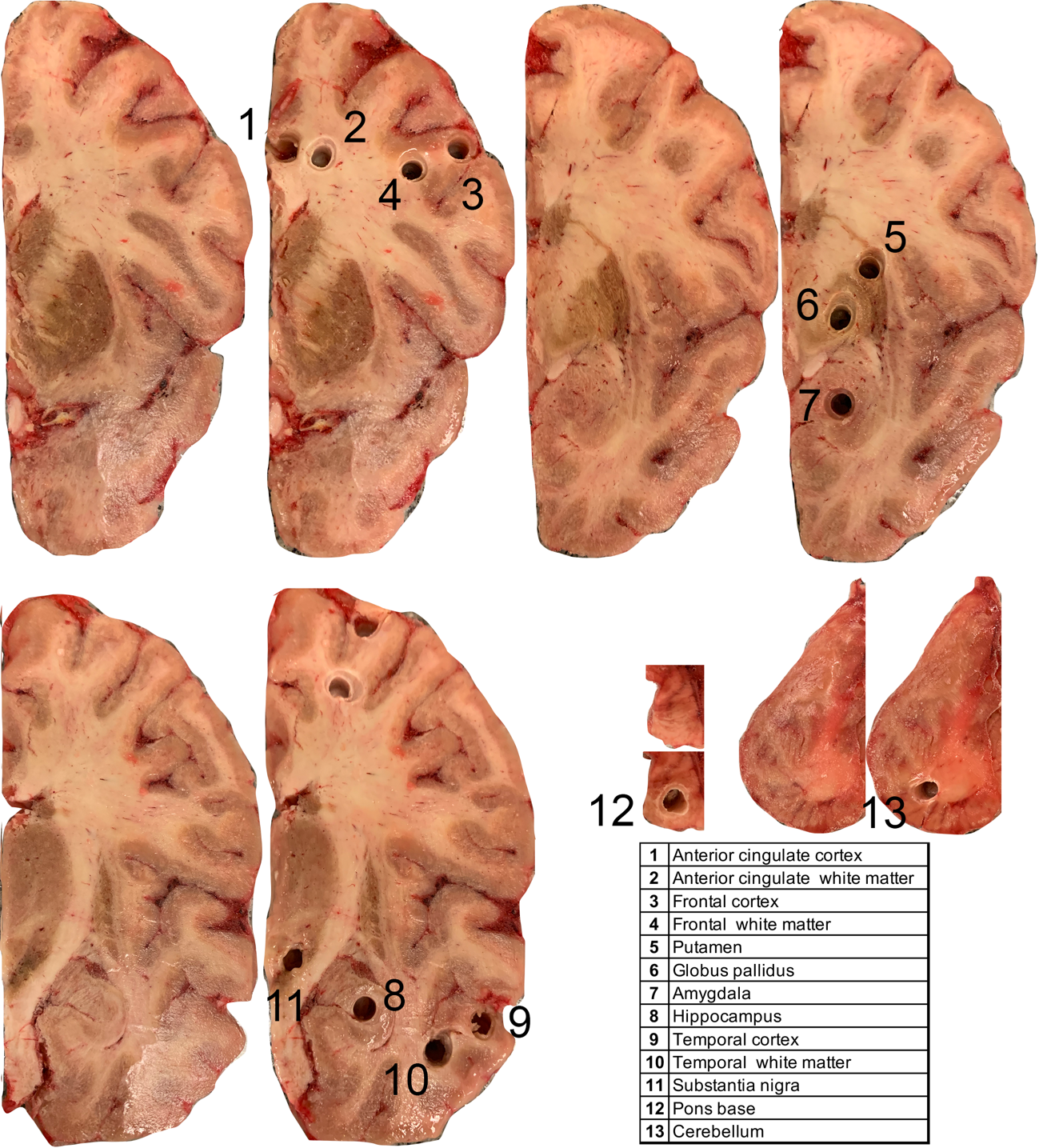
**

**Figure S4: Dissection of brain regions for protein extraction and α-synuclein seeding evaluation.** Coronal sections from frozen brains were used to dissect, up to 13 different brain regions in all the subjects included in the study, using a 4-mm brain tissue punch biopsy tool. Different biopsy punch tools were used between subjects and regions to avoid cross-contamination. Once the brain regions were dissected, they were stored at -80ºC until the protein was extracted.

**
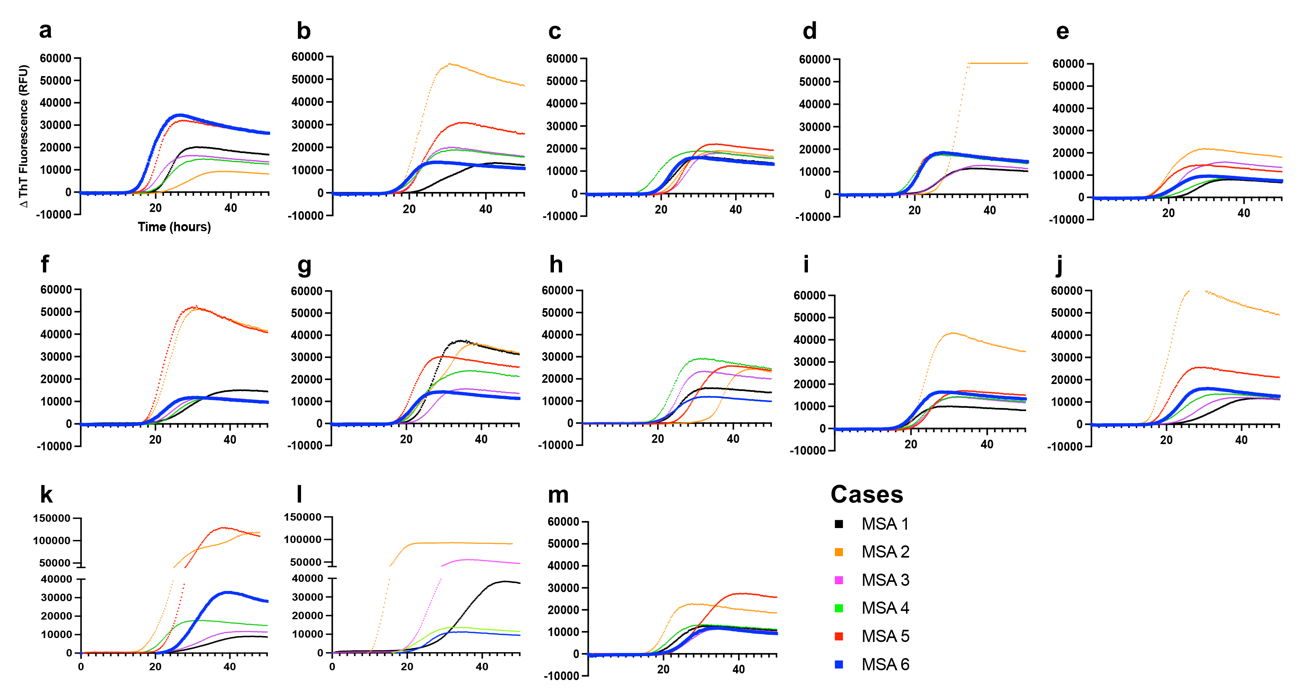
**

**Figure S5: The inter-individual α-synuclein seeding and intra-individual α-synuclein seeding behavior is distinct between MSA patients and brain regions.** Kinetic curves of α-synuclein seeding activity measured by RT-QuIC in **a)** anterior cingulate cortex **b)** anterior cingulate white matter **c)** frontal cortex **d)** frontal white matter **e)** putamen **f)** globus pallidus **g)** amygdala **h)** hippocampus **i)** temporal cortex **j)** temporal white matter **k)** substantia nigra **l)** pons base and **m)** cerebellum white matter from 6 different MSA patients. Each curve depicts the average of quadruplicates. Standard deviation (SD) was hidden to make the image more readable.

**
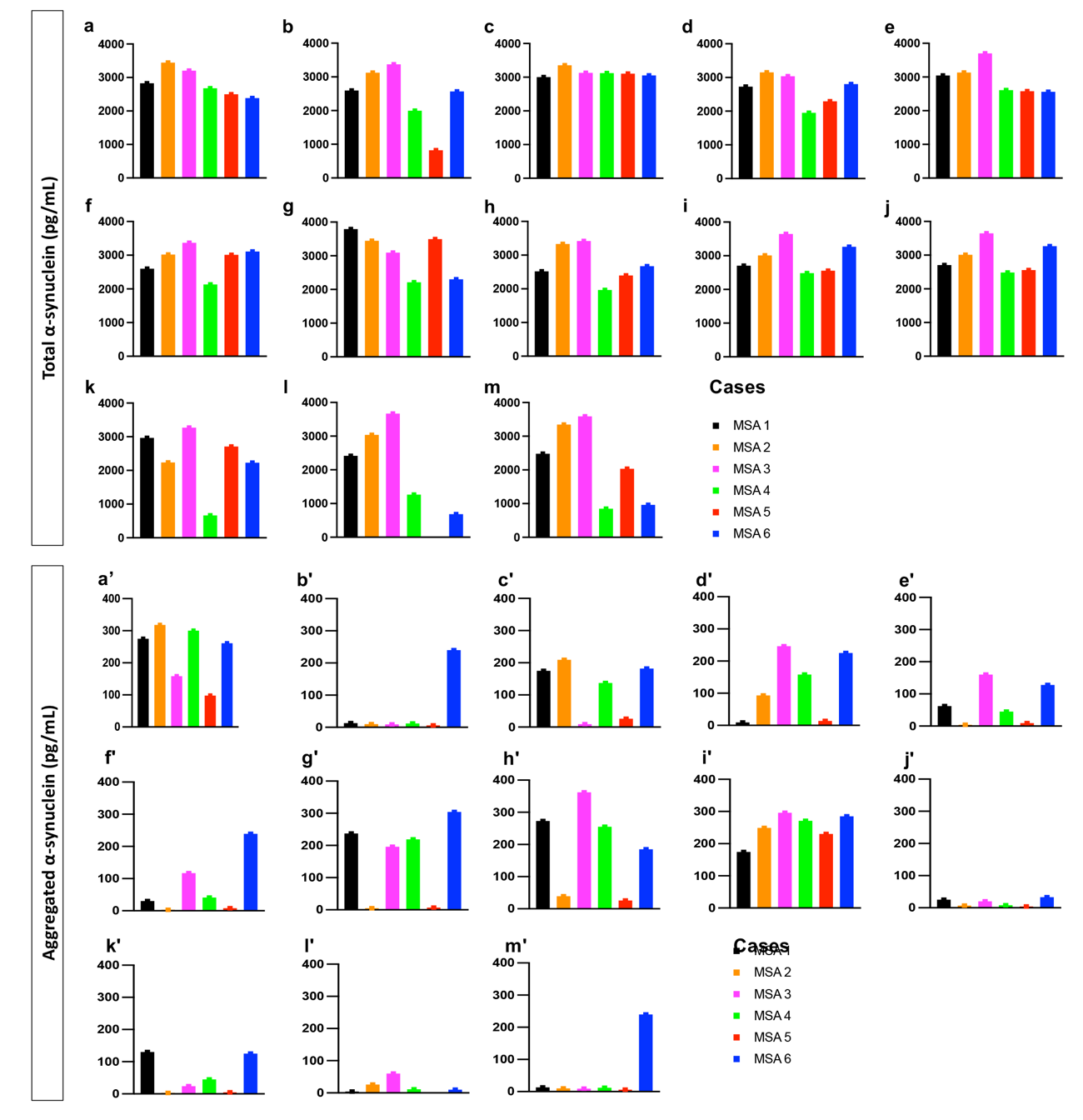
**

**Figure S6: The levels of total** α**-synuclein in each brain region are relatively uniform but the burden of aggregated α-synuclein varies across patients and brain regions in MSA patients.** The amount of total α-synuclein was quantified, using an ELISA assay, in **a)** anterior cingulate cortex **b)** anterior cingulate white matter **c)** frontal cortex **d)** frontal white matter **e)** putamen **f)** globus pallidus **g)** amygdala **h)** hippocampus **i)** temporal cortex **j)** temporal white matter **k)** substantia nigra **l)** pons base and **m)** cerebellar white matter from 6 different MSA patients. The amount of aggregated α-synuclein was quantified using the α-synuclein Patho ELISA assay, that uses the 5G4 antibody as capture antibody, in the same regions **a’)** anterior cingulate cortex **b’)** anterior cingulate white matter **c’)** frontal cortex **d’)** frontal white matter **e’)** putamen **f’)** globus pallidus **g’)** amygdala **h’)** hippocampus **i')** temporal cortex **j’)** temporal white matter **k')** substantia nigra **l’)** pons base and **m’)** cerebellar white matter from 6 different MSA patients

**
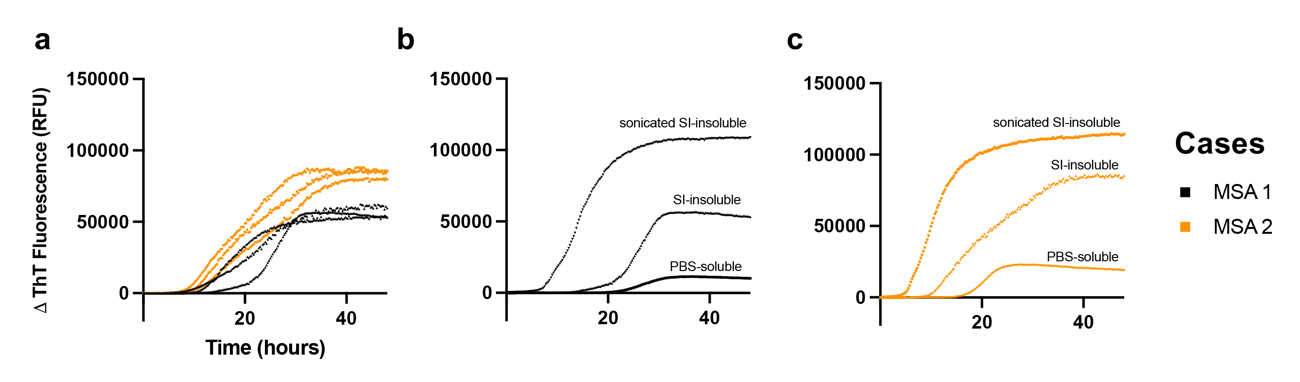
**

**Figure S7: The inter-individual but not the intra-individual α-synuclein seeding heterogeneity is preserved using the sarkosyl insoluble fraction.** Kinetic curves of α-synuclein seeding activity measured by RT- QuIC of a) the sarkosyl-insoluble fraction (SI) of the cerebellum, putamen and frontal cortex from two MSA cases b) the sonicated SI-insoluble, non-sonicated SI-insoluble and PBS-soluble fractions from the cerebellum of the MSA 1 c) the sonicated SI-insoluble, non-sonicated SI-insoluble and PBS-soluble fractions from the cerebellum of the MSA 2.

**
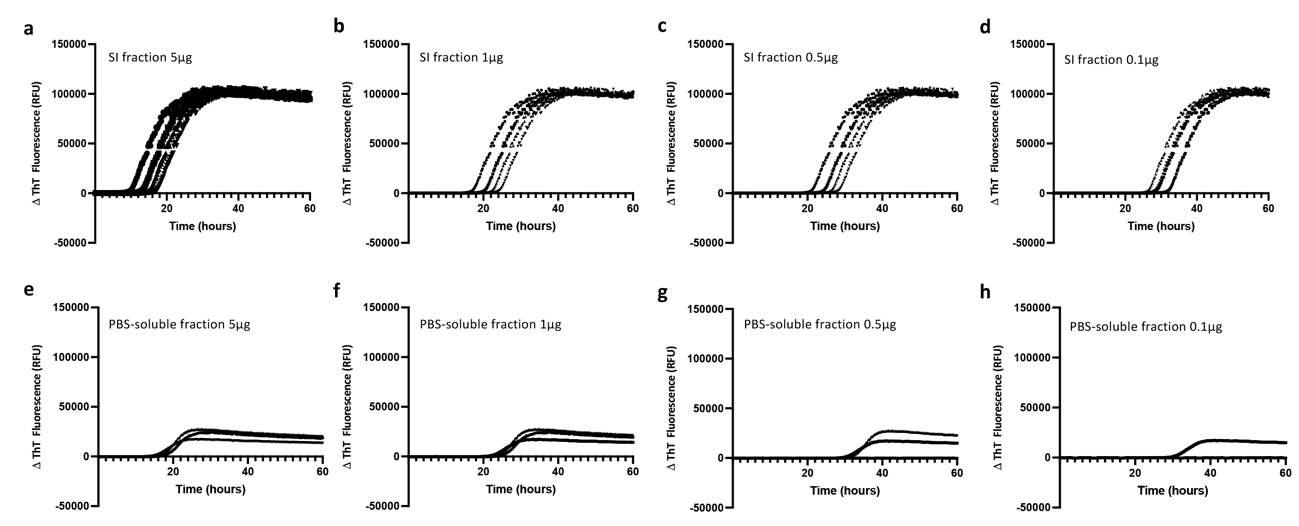
**

**Figure S8: The SI fraction promotes a faster aggregation and reaches a higher fluorescence plateau than the PBS-soluble fraction.** End-point dilution of the cerebellar sarkosyl-insoluble (SI) fraction and the PBS-soluble fraction from the MSA#3 subject. Kinetic curves of α-synuclein seeding activity measured by RT-QuIC of **a)** 5 μg, **b)** 1 μg, **c)** 0.5 μg and **d)** 0.1 μg of the SI fraction and of **e)** 5 μg, **f)** 1 μg, **g)** 0.5 μg and **h)** 0.1 μg of the PBS-soluble fraction from the cerebellum of the MSA#3 subject. Each curve depicts each of the four replicates made per condition.


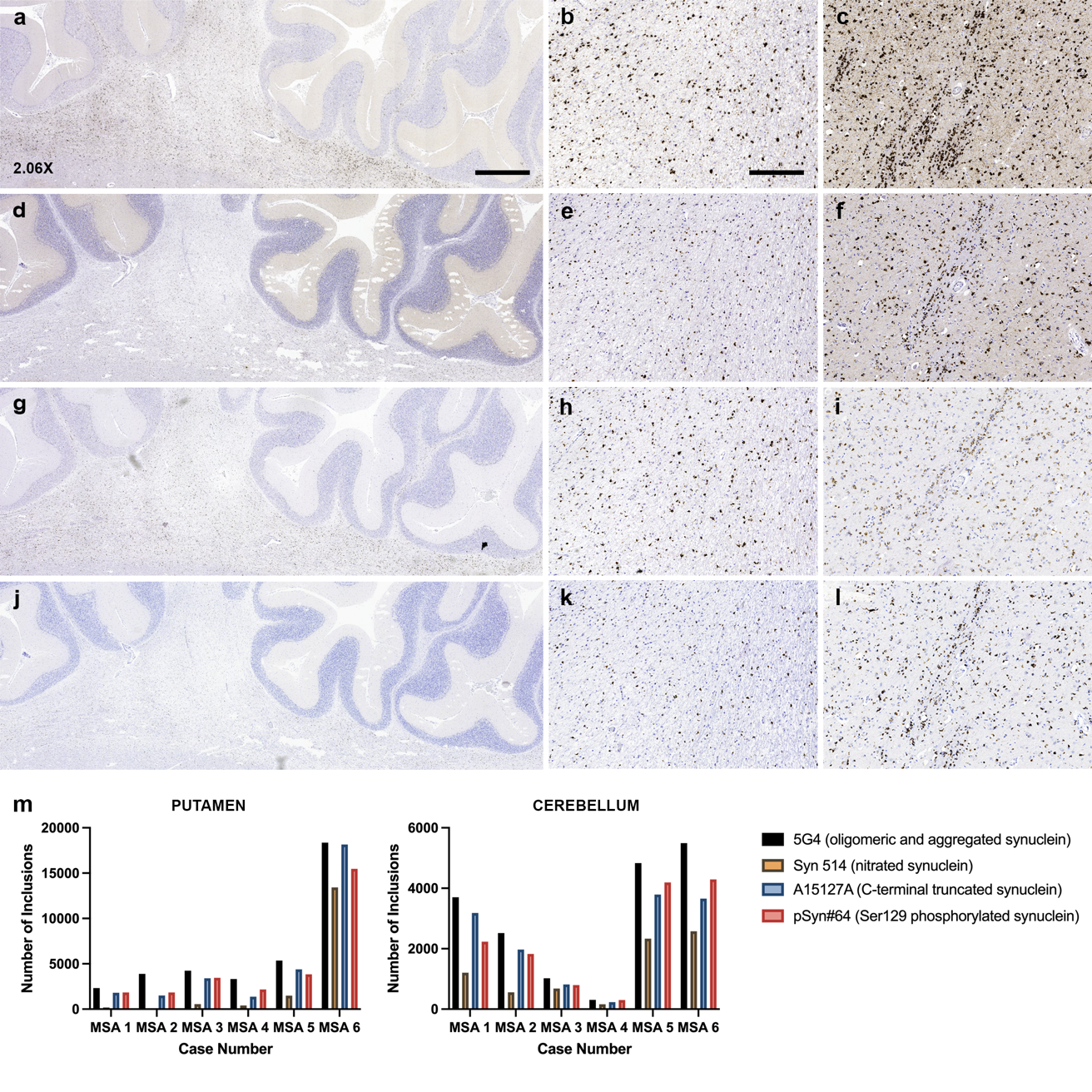


**Figure S9: The extent of** **pathology detected by different** α**-synuclein antibodies is not uniform.** Representative images showing **a)** 5G4 immunoreactivity in the cerebellum of MSA 6 at low magnification (2.06X); **b-c)** 5G4 immunoreactivity in the cerebellum and putamen of MSA 6 at higher magnification (6.25X), respectively; **d)** immunoreactivity showing nitrated α-synuclein inclusions in the cerebellum of MSA 6 at low magnification (2.06X); **e-f)** nitrated α-synuclein inclusions in the cerebellum and putamen of MSA 6 at higher magnification (6.25X), respectively; **g)** immunoreactivity showing truncated α-synuclein inclusions in the cerebellum of MSA 6 captured at low magnification (2.06X); **h-i)** truncated α-synuclein inclusions in the cerebellum and putamen of MSA 6 at higher magnification (6.25X), respectively. **j)** Ser 129 phosphorylated α-synuclein immunoreactivity in the cerebellum of MSA 6 at low magnification (2.06X); **k-l)** phosphorylated α-synuclein inclusions in the cerebellum and putamen of MSA 6 at higher magnification (6.25X), respectively; **m)** number of inclusions observed using the 4 different antibodies against α-synuclein in the putamen and cerebellum of 6 MSA patients. Scanned images were cropped at the same region to match the location in the images taken from different α-synuclein antibodies at the same magnification and size. Scale bar in a represents 4000 μm in a, d, g, j and scale bar represents 125 μm in b, c, e, f, h, i, k, l.

**
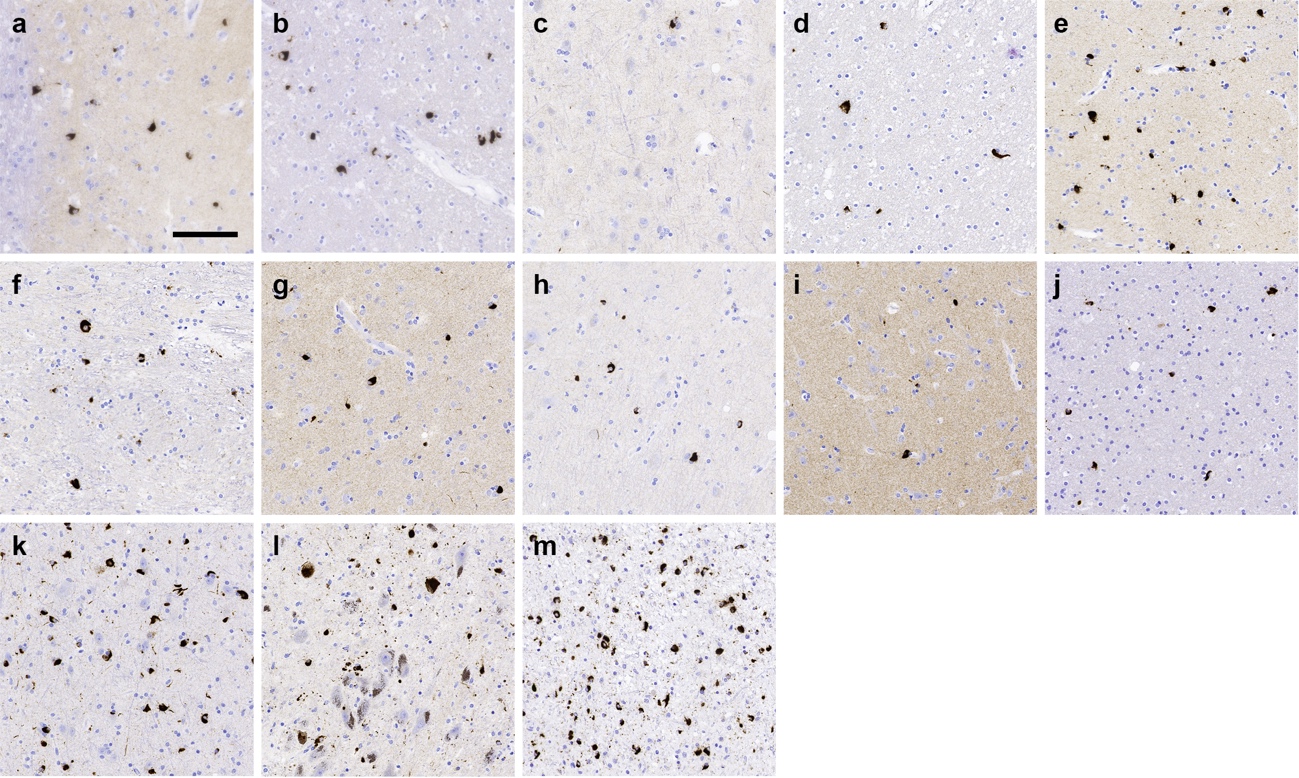
**

**Figure S10: GCIs and NCIs deposition across different brain regions in MSA patients.** Representative immunohistochemistry images for aggregated α-synuclein in **a)** anterior cingulate cortex **b)** anterior cingulate white matter **c)** frontal cortex **d)** frontal white matter **e)** putamen **f)** globus pallidus **g)** amygdala **h)** hippocampus **i)** temporal cortex **j)** temporal white matter **k)** substantia nigra **l)** pons base and **m)** cerebellum white matter from the MSA5 patient. Scale bar in ‘**a**’= 50 μm (applies to all images).

**
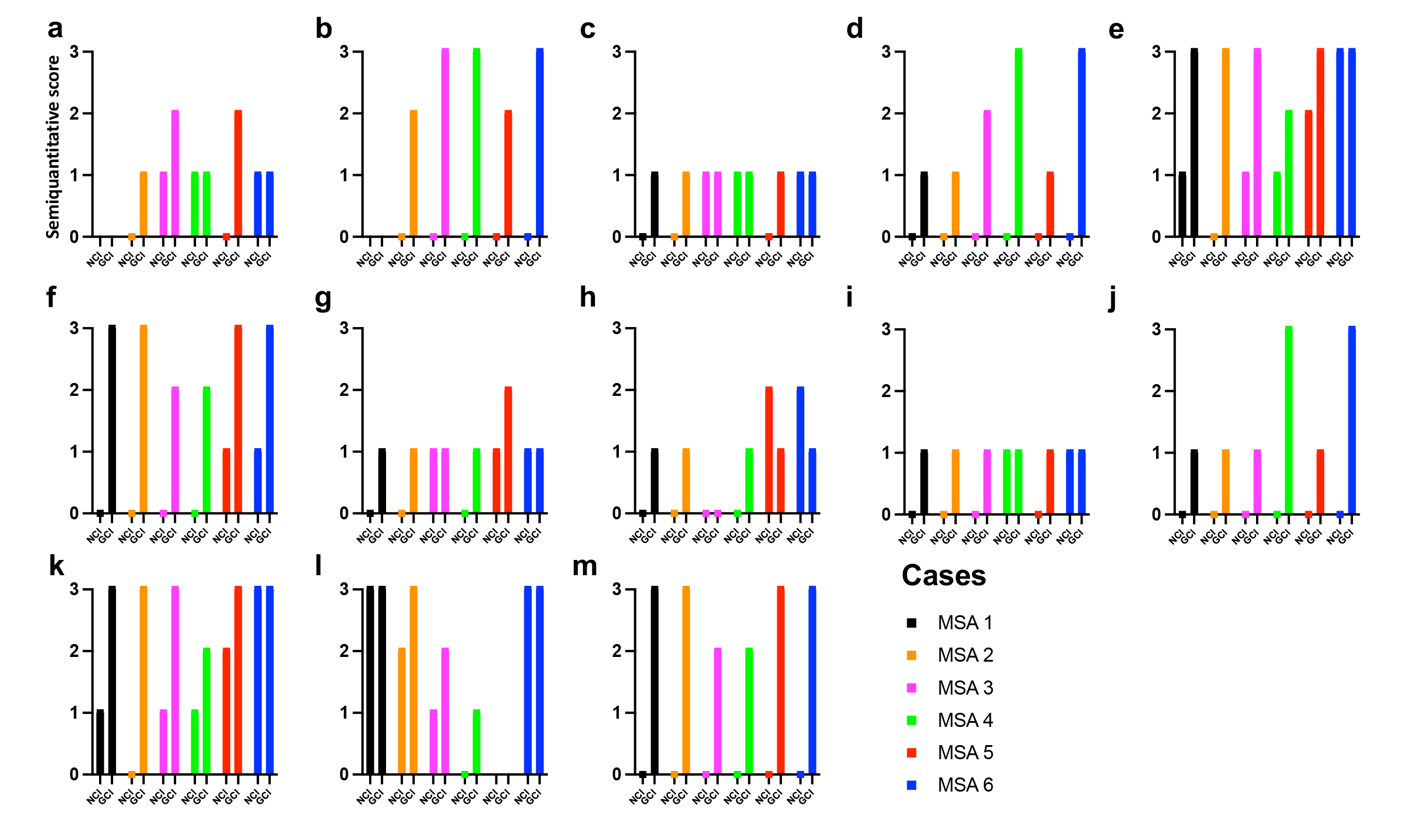
**

**Figure S11: The burden of GCIs and NCIs varies across different brain regions in MSA.** The burden of GCIs (second bar) and NCIs (first bar) was evaluated using a semiquantitative 4-point scale where 0 was the absence of α-synuclein pathology; 1 was mild; 2 was moderate and 3 was severe. The evaluation was performed in the **a)** anterior cingulate cortex **b)** anterior cingulate white matter **c)** frontal cortex **d)** frontal white matter **e)** putamen **f)** globus pallidus **g)** amygdala **h)** hippocampus **i)** temporal cortex **j)** temporal white matter **k)** substantia nigra **l)** pons base and **m)** cerebellum white matter from 6 different MSA patients.

**Table S1. Demographic and neuropathological diagnosis of the subjects included in this study.**

| **Case** | **Code** | **Source** | **Sex** | **Age** | **NIA-AA** | **Cerebral Amyloid Angiopathy** | **Lewy Body Pathology** | **Lewy Body Braak Stage** | **LATE-NC Stage** | **Other Pathologic Diagnosis** |
| --- | --- | --- | --- | --- | --- | --- | --- | --- | --- | --- |
| **LBD 1** | 561 | Canada | F | 59 | A3B3C3 | - | Neocortical (diffuse) | Stage 5 | - | - |
| **LBD 2** | 973 | Canada | F | 82 | A2B3C3 | Aβ-positive Type 2 | Neocortical (diffuse) | Stage 5 | Stage 2 | - |
| **LBD 3** | 1074 | Canada | M | 73 | A2B3C2 | Aβ-positive Type 2 | Neocortical (diffuse) | Stage 5 | Stage 2 | - |
| **LBD 4** | 1075 | Canada | M | 71 | A2B2C3 | - | Neocortical (diffuse) | Stage 5 | - | - |
| **LBD 5** | 1153 | Canada | M | 79 | A3B3C3 | Aβ-positive Type 1 and 2 | Neocortical (diffuse) | Stage 5 | - | - |
| **LBD 6** | 1160 | Canada | M | 84 | A2B2C2 | Aβ-positive Type 1 | Limbic (transitional) | Stage 4 | Not Assessed | - |
| **LBD 7** | 1181 | Canada | F | 78 | A3B3C3 | - | Neocortical (diffuse) | Stage 5 | Stage 2 | - |
| **LBD 8** | 1195 | Canada | M | 80 | A2B3C2 | Aβ-positive Type 1 and 2 | Limbic (transitional) | Stage 4 | Stage 1 | - |
| **LBD 9** | 1245 | Canada | F | 82 | A3B3C3 | Aβ-positive Type 2 | Limbic (transitional) | Stage 4 | Stage 2 | - |
| **LBD 10** | 1239 | Canada | M | 73 | A2B2C2 | - | Neocortical (diffuse) | Stage 5 | - | - |
| **LBD 11** | 1265 | Canada | F | 88 | A2B2C2 | Aβ-positive Type 1 and 2 | Limbic (transitional) | Stage 4 | Stage 2 | - |
| **LBD 12** | 1305 | Canada | F | 94 | A2B2C2 | Aβ-positive Type 2 | Neocortical (diffuse) | Stage 5 | - | - |
| **LBD 13** | 1404 | Canada | F | 73 | A2B3C2 | - | Limbic (transitional) | Stage 4 | Stage 2 | - |
| **LBD 14** | 1409 | Canada | F | 76 | A3B3C3 | Aβ-positive Type 2 | Neocortical (diffuse) | Stage 5 | Stage 2 | - |
| **LBD 15** | 1424 | Canada | M | 69 | A1B1C0 | - | Limbic (transitional) | Stage 4 | - | - |

| **Case** | **Code** | **Source** | **Sex** | **Age** | **NIA-AA** | **Cerebral Amyloid Angiopathy** | **Lewy Body Pathology** | **Lewy Body Braak Stage** | **LATE-NC Stage** | **Other Pathologic Diagnosis** |
| --- | --- | --- | --- | --- | --- | --- | --- | --- | --- | --- |
| **MSA 1** | 585 | Canada | F | 68 | A1B1C0 | Aβ-positive Type 2 | - | - | - | ARTAG Medial Temporal Lobe WM |
| **MSA 2** | 1381 | Canada | M | 72 | A1B0C0 | - | - | - | - | Hemorrhage: Pons Base & Thalamus |
| **MSA 3** | 1461 | Canada | F | 76 | A0B1C0 | - | - | - | - | PART (Braak stage II) |
| **MSA 4** | R-NBC-20-31 | Canada | M | 61 | A0B1C0 | - | - | - | - | PART (Braak stage II) |
| **MSA 5** | R-NBC-20-9 | Canada | M | 64 | A0B1C0 | - | - | - | - | PART (Braak stage II), AGD (Stage II) |
| **MSA 6** | R-NBC-20-19 | Canada | M | 62 | A0B1C1 | - | - | - | - | ARTAG Medial Temporal Lobe GM |
| **MSA 7** | 1613 | Canada | M | 62 | A0B1C0 | - | - | - | - | - |
| **MSA 8** | 1704 | Canada | M | 73 | A0B1C0 | - | - | - | - | - |
| **MSA 9** | A-14-02 | Canada | M | 71 | A0B1C0 | - | - | - | - | - |
| **MSA 10** | BCN-306 | Spain | F | 65 | A0B1C0 | - | - | - | - | - |
| **MSA 11** | BCN-321 | Spain | M | 77 | A0B1C0 | - | - | - | - | - |
| **MSA 12** | BCN-371 | Spain | F | 80 | A0B1C0 | - | - | - | - | - |
| **MSA 13** | BCN-391 | Spain | M | 75 | A2B1C1 | - | - | - | - | - |
| **MSA 14** | BCN-427 | Spain | F | 81 | A2B1C2 | - | - | - | - | - |
| **MSA 15** | BCN-480 | Spain | M | 70 | A0B1C0 | - | - | - | - | - |

| **Case** | **Code** | **Source** | **Sex** | **Age** | **NIA-AA** | **Cerebral Amyloid Angiopathy** | **Lewy Body Pathology** | **Lewy Body Braak Stage** | **LATE-NC Stage** | **Other Pathologic Diagnosis** |
| --- | --- | --- | --- | --- | --- | --- | --- | --- | --- | --- |
| **PSP 1** | 1014 | Canada | F | 77 | - | Aβ-positive Type 2 | - | Amygdala only | - | - |
| **PSP 2** | 1443 | Canada | F | 74 | - | - | - | - | - | - |
| **PSP 3** | 1552 | Canada | F | 74 | - | - | - | - | - | - |
| **PSP 4** | R-NBC-19-1 | Canada | F | 70 | - | - | - | - | - | - |
| **PSP 5** | R-NBC-20-13 | Canada | M | 73 | - | Aβ-positive Type 2 | - | - | - | Argyrophilic Grain Disease (4R) |
|  |  |  |  |  |  |  |  |  |  |  |
| **Control 1** | 1051 | Canada | M | 74 | - | - | - | - | - | - |
| **Control 2** | 1095 | Canada | F | 48 | - | - | - | - | - | - |
| **Control 3** | 1113 | Canada | F | 69 | - | - | - | - | - | - |
| **Control 4** | 1466 | Canada | F | 53 | - | - | - | - | - | - |
| **Control 5** | 1509 | Canada | F | 26 | - | - | - | - | - | - |
